# Frequent asymmetric migrations suppress natural selection in spatially structured populations

**DOI:** 10.1093/pnasnexus/pgad392

**Published:** 2023-11-14

**Authors:** Alia Abbara, Anne-Florence Bitbol

**Affiliations:** Institute of Bioengineering, School of Life Sciences, École Polytechnique Fédérale de Lausanne (EPFL), CH-1015 Lausanne, Switzerland; SIB Swiss Institute of Bioinformatics, CH-1015 Lausanne, Switzerland; Institute of Bioengineering, School of Life Sciences, École Polytechnique Fédérale de Lausanne (EPFL), CH-1015 Lausanne, Switzerland; SIB Swiss Institute of Bioinformatics, CH-1015 Lausanne, Switzerland

## Abstract

Natural microbial populations often have complex spatial structures. This can impact their evolution, in particular the ability of mutants to take over. While mutant fixation probabilities are known to be unaffected by sufficiently symmetric structures, evolutionary graph theory has shown that some graphs can amplify or suppress natural selection, in a way that depends on microscopic update rules. We propose a model of spatially structured populations on graphs directly inspired by batch culture experiments, alternating within-deme growth on nodes and migration-dilution steps, and yielding successive bottlenecks. This setting bridges models from evolutionary graph theory with Wright–Fisher models. Using a branching process approach, we show that spatial structure with frequent migrations can only yield suppression of natural selection. More precisely, in this regime, circulation graphs, where the total incoming migration flow equals the total outgoing one in each deme, do not impact fixation probability, while all other graphs strictly suppress selection. Suppression becomes stronger as the asymmetry between incoming and outgoing migrations grows. Amplification of natural selection can nevertheless exist in a restricted regime of rare migrations and very small fitness advantages, where we recover the predictions of evolutionary graph theory for the star graph.

**Significance Statement**

The spatial structures of microbial populations are often complex. This can impact their evolution, in particular the ability of mutants to take over the population. We introduce a new model of spatially structured populations on graphs, which is directly inspired by batch culture experiments. We show that for frequent migrations, suppression of natural selection is pervasive when there is asymmetry between the total incoming and outgoing migration flows to and from a deme. If there is no such asymmetry, spatial structure does not impact mutant fixation probability. Amplification of natural selection can only exist for very small fitness advantages, in a restricted regime of rare migrations. Our results reconcile and generalize previous ones, and allow for direct experimental tests.

## Introduction

Natural microbial populations often present complex spatial structures, where not all organisms are in equal competition. For instance, populations of pathogens are subdivided between different organs during infections (1, 2) and evolve within each host during epidemics (3), commensal bacteria are spread through the gut (4) where they evolve (5, 6), and ecosystems are shaped by local resources (7). Even well-agitated liquid suspensions deviate from idealized well-mixed populations where all organisms are in equal competition (8). To incorporate spatial structure into population models, early works considered populations divided into several well-mixed subpopulations or demes, with possible migrations between them (9, 10). In particular, Maruyama showed that the fixation probability of a mutant is not impacted by spatial structure, under the assumption that migrations are sufficiently symmetric to preserve the overall mutant fraction (11, 12). Note, however, that even highly symmetric spatial structures can impact mutant fixation probability if extinctions of demes occur (13).

Evolutionary graph theory allows to model complex spatial structures (14). In this framework, one individual is located on each node of a graph, and replacement probabilities are specified along its edges. The state of the population evolves according to the Moran model (15) using a specific update rule. For instance, in the Birth–death update rule [also known as biased invasion process (16, 17)], an individual is first selected proportionally to its fitness to divide, and then its offspring replaces one of its neighbors on the graph. In the death–Birth update rule [also known as biased voter model (16, 17)], an individual is first selected uniformly at random to die, and then one of its neighbors on the graph is selected proportionally to fitness to divide, and sends its offspring to the empty node. Although these two rules seem very similar, choosing one or the other strongly impacts the evolutionary outcome (18, 19). For example, the star graph amplifies natural selection under the Birth–death update rule, but suppresses it under the death–Birth update rule (18, 20, 21). Evolutionary graph theory models have been generalized by placing well-mixed demes on graph nodes, rather than single individuals, also using the Moran model with update rules (17, 22–25). In all these models, population sizes are strictly constant, and birth and death events are coupled and occur in a specific order. Besides, migration events are either coupled to birth and death (17, 22–25) or independent from them but symmetric (25).

In natural microbial populations, the number of individuals is generally not strictly constant, even though it may be limited, e.g. by resource availability. Furthermore, there is no imposed order of individual birth and death events. Thus, to make a link with natural situations and with evolution experiments (26–33), a more universal theoretical description, whose results do not depend on microscopic update rules, is needed. We made a first step in this direction in Ref. (34), by considering independent events of birth, death, and migration, in a model where deme sizes could fluctuate around a steady-state value. When exchanges between demes are rare, we showed that the star graph can either amplify or suppress natural selection depending on the asymmetry between incoming and outgoing migrations to and from the center. However, the results of Ref. (34) are limited to the rare migration regime, where each deme can be considered as either fully mutant or fully wild-type upon migration events.

Here, we present a new model of spatially structured populations on graphs, directly inspired by the batch culture setups with serial transfers that are used in many evolution experiments (26–33), including those with spatially structured populations (29–31, 33). Our model is formally close to a structured Wright–Fisher model, and allows us to bridge classical population genetics models (9–13) with evolutionary graph theory (14, 18, 19). We investigate the impact of population structure on mutant fixation probability and fixation time. We consider frequent migrations between demes, which can result in mixed states of the demes. We find that in this regime, the star suppresses natural selection and accelerates evolutionary dynamics, provided there is asymmetry between incoming and outgoing migrations to and from the center. More generally, using a branching process approach, we demonstrate that with frequent migrations, all graphs strictly suppress natural selection compared to a well-mixed population, except circulation graphs, where the total incoming migration flow equals the total outgoing one in each deme. In this regime, circulation graphs have no impact on fixation probability. Stochastic simulations confirm our analytical predictions, and show that suppression of selection becomes stronger as the asymmetry between incoming and outgoing migrations grows. Amplification of natural selection can nevertheless exist in a restricted regime of rare migrations, where we recover the results of Ref. (34) and the predictions of evolutionary graph theory for the star.

## Results

### Deme-structured populations with serial dilutions

**Fig. 1.**
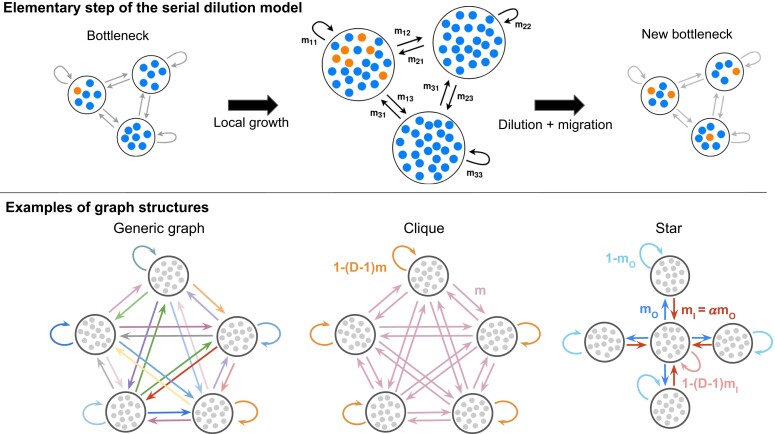
Schematic of our model and of some graph structures. Top: one elementary step of the serial dilution model for structured populations. Starting from a bottleneck, demes first undergo a phase of local growth. Then, dilution and migration occur along the edges of the graph, according to migration probabilities. A new bottleneck state is reached. Bottom: examples of graph structures for D=5 demes. From left to right: a generic graph with various migration probabilities, and two strongly symmetric graphs. For the clique, all migrations probabilities between different demes are equal to *m*. It corresponds to Wright’s island model (9). For the star, the outgoing migration probability from the center to a leaf is $m_{O}$, while the incoming migration probability from a leaf to the center is $m_{I}=\alpha m_{O}$.

To model population spatial structure, we consider *D* demes on the nodes of a connected graph with two types of individuals: wild-types with fitness $f_{W}=1$, and mutants with fitness $f_{M}=1+s$. Fitnesses represent division rates during stages of exponential growth. We propose a model with serial phases of exponential growth and dilution (see Methods section for details). This model is highly relevant to describe evolution experiments in batch culture with serial transfers (26–29, 32), including experiments with controlled spatial structures (29–31, 33). In addition, it is formally close to the Wright–Fisher model, allowing us to connect with classical results. For simplicity, individuals are assumed to be haploids that reproduce asexually, but generalizations could be made beyond this case, focusing on a single locus, as in the Wright–Fisher model. The key elementary steps are the following (see Fig. 1, top panel). Each deme undergoes exponential growth for time *t*, reaching a large size from an initial bottleneck size. Then, binomial sampling is performed from each deme *i* to each deme *j* (including j=i) so that on average $Km_{ij}$ individuals are transferred from the grown deme *i* to form the next bottleneck of deme *j*. Here $m_{ij}$ denotes the probability to migrate from deme *i* to deme *j* at a sampling step. Sampling corresponds to dilution and migration. We assume $\sum_{i}m_{ij}=1$, so the typical bottleneck size of all demes is *K*. The case $\sum_{j}m_{ij}=1$, where each deme typically contributes by the same amount *K* to the next bottleneck of the population, is also discussed in the Supplementary material. This two-step process of growth and dilution-migration is then repeated, until one of the two types of individuals fixes.

We investigate fixation probability and fixation time in this model. In particular, we ask about the impact of the structure of the graph on these quantities. We consider generic graphs with various migration probabilities, and we give specific results for strongly symmetric graphs, including the clique and the star (see Fig. 1, bottom panel).

### With frequent migrations, the star suppresses natural selection and accelerates evolutionary dynamics

The star has been intensely studied in evolutionary graph theory, and is an amplifier of natural selection in the Birth–death process but a suppressor in the death–Birth process (14, 16, 18, 20, 21). An amplifier of selection yields a higher fixation probability than a well-mixed population for beneficial mutants, and a lower one for deleterious mutants, while a suppressor of selection does the opposite. How does the star impact mutant fixation in our model, which does not rely on an update rule, and where a well-mixed deme sits on each node of the graph? We denote by $\alpha =m_{I}/m_{O}$ the asymmetry between incoming probabilities $m_{I}$ and outgoing migration probabilities $m_{O}$ between the center and the leaves (see Fig. 1). In the restricted regime of rare migrations, we previously showed that migration asymmetry determines whether the star is a suppressor (for α<1) or an amplifier (for α>1) of selection (34). Here, we consider the more general case of frequent migrations.

**Fig. 2.**
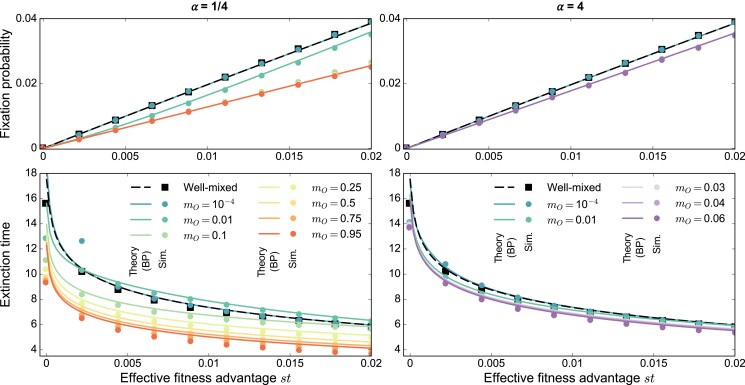
Mutant fixation and extinction in the star. Mutant fixation probability (top) and average extinction time (bottom, in numbers of dilution steps) are plotted versus the effective fitness advantage *st* of the mutant. We consider a star with D=5 demes, and K=1,000 individuals per deme on average at the bottleneck, for migration asymmetries α=1/4 (left) and α=4 (right). We start with one mutant of fitness $f_{M}=1+s$ placed uniformly at random at a bottleneck, all other individuals being wild-types with fitness $f_{W}=1$. The growth phase duration is t=5. Markers represent simulation results (“Sim.”), averaged on 1 million realizations. Lines are theoretical predictions from our branching process (“BP”) approach (see Section 4.2 in the Supplementary Material for fixation probabilities and Supplementary Eq. S31 for extinction times). The well-mixed case is shown for comparison, with simulations performed for a population with KD=5,000 individuals at the bottleneck, initialized with one mutant. For the star, the outgoing migration probability $m_{O}$ ranges between $10^{-4}$, above the rare migration regime ($≲10^{-6}$, see Supplementary Section 2, esp. Eq. S17, in the Supplementary Material), and values close to 1. Note that for α=1/4, $m_{O}$ can vary between 0 and 1, while for α=4, $m_{O}$ is constrained to a smaller range (see Supplementary material).

Starting with one single mutant placed uniformly at random in a deme at a bottleneck, what is its fixation probability? The coarse-grained description valid for rare migrations, where each deme is either fully mutant or fully wild-type (34, 35), cannot be used for more frequent migrations. We develop a multitype branching process approach, which holds when deme size *K* is large, while the effective fitness advantage *st* is positive and small, but larger than 1/K, and for nonrare migrations, see Methods section. In what follows, we will refer to this parameter regime as the branching process regime. For the star, we obtain the fixation probability $\rho_{C}$ (resp. $\rho_{L}$) starting from a mutant placed in the center (resp. in a leaf) at a bottleneck, as well as their average $\rho =[\rho_{C}+(D-1)\rho_{L}]/D$ for a randomly placed mutant, to first order in *st* (see Supplementary Section 3.3.2). We find that $\rho_{L}=\alpha \rho_{C}$: for α>1, the mutant is more likely to fix starting from a leaf than from the center, and conversely when α<1 (see Supplementary Fig. S1). More precisely, α<1 means that $m_{O}>m_{I}$, which makes mutants in the center spread easily to the leaves, giving the center an advantage—but mutants are more likely to start from the leaves. Moreover, we find that ρ≤2st in all cases, where 2st is the fixation probability in a well-mixed population (36). Thus, the star always suppresses natural selection in this regime. Figure 2 (top panels) shows both analytical predictions and simulation results, in excellent agreement. We observe that while the fixation probability in the star is close to the well-mixed one for relatively rare migrations, suppression becomes stronger as migrations become more frequent.

The average time for mutants to go extinct (conditioned on extinction) can also be derived in our branching process approach (see Methods section). We find that extinction becomes faster in the star than in a well-mixed population when migrations are strong. Figure 2 (bottom panels) shows excellent agreement between theory and simulations, except for very small values of *st*, which are outside the range of validity of our branching process approach. When migration probabilities are small, exchanges between demes are slow and extinction takes longer than in a well-mixed population. However, as migration probabilities reach $m_{O}=0.1$ and above, extinction times become shorter. For such frequent migrations, simulations further reveal a faster growth of mutant fraction in trajectories leading to fixation, and shorter average fixation times, in the star versus the well-mixed population (see Supplementary Figs. S2 and S3). This is associated to the lower fixation probability in the star: mutants either grow fast enough to survive fluctuations and reach fixation, or go extinct. These results stand in contrast with those of evolutionary graph theory under the Birth–death process, where spatial structure is generally found to slow down fixation compared to the well-mixed case (37–39). Our model shows such a slowdown of fixation for rarer migrations, in line with expectations (see Supplementary Figs. S2 and S3).

Qualitatively similar conclusions are obtained if each deme typically contributes by the same amount *K* to the next bottleneck ($\sum_{j}m_{ij}=1$ for all *i*), see Fig. S4 in the Supplementary Material. Moreover, different sampling schemes for bottlenecks yield the same results in the branching process regime, see Fig. S5 in the Supplementary Material. This shows the robustness of our conclusions for the star in the branching process regime for strong migrations.

### Asymmetry between incoming and outgoing migrations for each deme favors suppression of selection

Within the branching process approach, and for frequent migrations, we prove that no graph gives a higher fixation probability than the well-mixed population for randomly placed beneficial mutants (see Supplementary Section 3.1.2). In other words, spatial structure cannot amplify natural selection in this regime. Note that simulations show that suppression is still prominent when the fitness advantage of the mutant grows beyond the branching process regime.

The only graphs that do not strictly suppress natural selection for frequent migrations in the branching process regime are such that for each deme, the sum of incoming migration probabilities is equal to the sum of outgoing probabilities (see Supplementary Sections 3.4 and 3.6). This type of graph is called a *circulation*. Some examples are the clique, and the star with α=1, see Fig. 1, bottom. Remarkably, all circulations have the same probability of fixation as well-mixed populations within the branching process approach for frequent migrations (namely, 2st to first order in *st*, see Supplementary Section 3.4). This generalizes the circulation theorem of Ref. (14) which holds for graphs with one individual per node, as well as our extension (34) to graphs with one deme per node in the rare migration regime. Maruyama’s pioneering work showed that fixation probabilities are unaffected by spatial structure provided that migration does not change overall mutant frequency (11, 12). This is also known as conservative migration (40). Within our model, since all demes have the same average bottleneck size, i.e. $\forall j,\;\sum_{i}m_{ij}=1$, this amounts to requiring that the migration-dilution step preserves the average mutant frequencies in each deme, i.e. $x_{i}^{\prime }=\sum_{k}m_{ki}x_{k}^{\prime }$, for all possible values of the postgrowth mutant fractions $x_{i}^{\prime }$. This yields $\forall j,\;\sum_{i}m_{ji}=1=\sum_{i}m_{ij}$, which corresponds to circulations. Therefore, Maruyama’s theorem and the circulation theorem are two faces of a more general result.

**Fig. 3.**
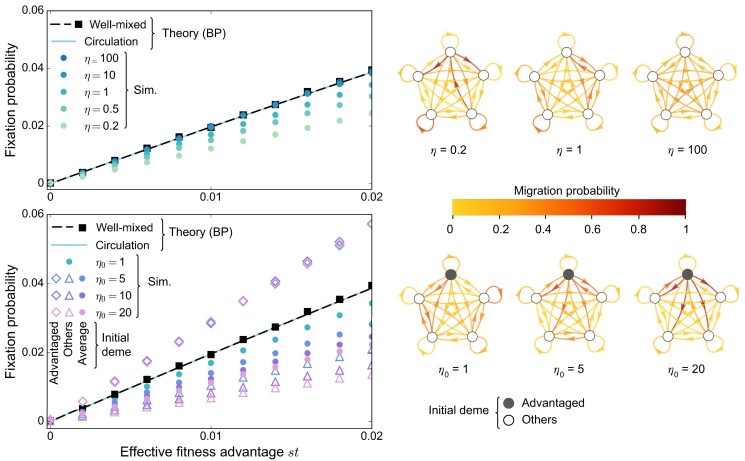
Mutant fixation probabilities for different asymmetries between incoming and outgoing migrations. Fixation probabilities are shown versus effective mutant fitness advantage *st* for Dirichlet cliques (see Supplementary Section 6) with D=5 demes, and K=1,000 individuals per deme on average at the bottleneck. We start with one mutant of fitness $f_{M}=1+s$ at a bottleneck, all other individuals being wild-types with fitness $f_{W}=1$. The growth phase duration is t=5. Markers represent simulation results (“Sim.”), averaged on 1 million realizations, with different Dirichlet cliques for each of them. The well-mixed case is shown for comparison, with simulations performed for a well-mixed population with KD=5,000 individuals at the bottleneck, initialized with one mutant. Lines are theoretical predictions from our branching process (“BP”) approach, for the well-mixed population and for a circulation with frequent migrations (see Supplementary Section 4.2). Top: We sample incoming migration probabilities $m_{ij}$ for each destination deme *j* from the same Dirichlet distribution, using the same parameter *η* for all origin demes. The average value of each $m_{ij}$ is 1/5, but their variances decrease as *η* increases. Examples of generated graphs are shown on the right. Mutants are placed uniformly at random. Bottom: We sample incoming migration probabilities $m_{ij}$ for each destination deme *j* from a Dirichlet distribution, with parameter $\eta_{0}\geq 1$ for one advantaged deme, and 1 for others. Examples are shown on the right. When $\eta_{0}=1$, all demes are equivalent, recovering the case η=1 in the top panel. As $\eta_{0}$ grows, the outgoing migration probabilities from the advantaged deme become larger. Fixation probabilities are shown when the initial mutant is placed in the advantaged deme, in another deme, and in a deme chosen uniformly at random (“Average”).

By contrast, any graph that is not a circulation is a strict suppressor of selection to first order in *st* for frequent migrations in the branching process regime (see Supplementary Section 3.6). Indeed, the fixation probability *ρ* averaged over the initial deme *i* of the mutant satisfies ρ<2st. Graphs deviate from circulations when total incoming and outgoing migrations differ. How does such migration asymmetry impact mutant fixation probability? To investigate this, we consider graphs where this asymmetry can be tuned. Specifically, we generate graphs that we call *Dirichlet cliques*, by sampling all incoming migration probabilities to a given deme *j* from a Dirichlet distribution, ensuring $\sum_{i}m_{ij}=1$ (see Supplementary Section 6). First, we take the same Dirichlet distribution for each destination deme *j*, with all parameters being equal to *η*. All migration probabilities $m_{ij}$ are then centered around the same value, but their variances are tuned by the parameter *η*. When *η* is small, migration probabilities have very contrasted values, while they become more homogeneous as *η* grows. A small *η* creates unbalance between the incoming and outgoing migration probabilities for each deme. Figure 3 (top panel) shows that Dirichlet cliques generated with large *η* have fixation probabilities very close to those of circulations. As *η* decreases, the probability of fixation in these graphs also decreases on average. Therefore, asymmetry between incoming and outgoing migrations is key to suppression of natural selection, and more asymmetry yields more suppression.

What happens if one specific deme sends more individuals to other demes than it receives from them? To address this question, we consider Dirichlet cliques where one special deme has a parameter $\eta_{0}\geq 1$, while all others have η=1 (see Supplementary Section 6). Then, $\eta_{0}$ quantifies the advantage of the special deme: as $\eta_{0}$ grows, the average value of outgoing migrations from the advantaged deme increases. Consequently, exchanges in the rest of the Dirichlet clique decrease, since $\sum_{i}m_{ij}=1$ for all *j*. Figure 3 (bottom panel) shows that the fixation probability for a mutant starting in the advantaged deme is higher than in a well-mixed population. Indeed, mutants in the advantaged deme can easily spread. Conversely, the spread and thus the fixation of a mutant placed in any other deme is hindered. Averaging over demes, we find that the fixation probability of a mutant placed uniformly at random is smaller than in a well-mixed population. These results generalize those we obtained for the star, where the center is an advantaged deme if α<1 (see above and Fig. S1 in the Supplementary Material). Moreover, a stronger unbalance between migration probabilities leads to more suppression of natural selection (see Fig. 3, bottom panel).

We proved that spatial structure suppresses selection for frequent asymmetric migrations, when mutants are initially placed uniformly at random. In addition, we showed that mutants starting in advantaged demes, which send more individuals to other demes than they receive from them, are more likely to fix than in a well-mixed population, while the opposite holds for other demes. Averaging over all demes can only yield suppression of selection. This is due to the nonlinearity (technically to the convexity) of the generating function of the branching process, from which extinction probabilities are derived (see Supplementary Section 3.1.2). Note that the acceleration of mutant extinction in the star versus the well-mixed population (see Fig. 2) arises similarly: a slowdown for advantaged demes and an acceleration for others result in an overall acceleration.

### Amplification can happen for rare migrations and weakly beneficial mutants

**Fig. 4.**
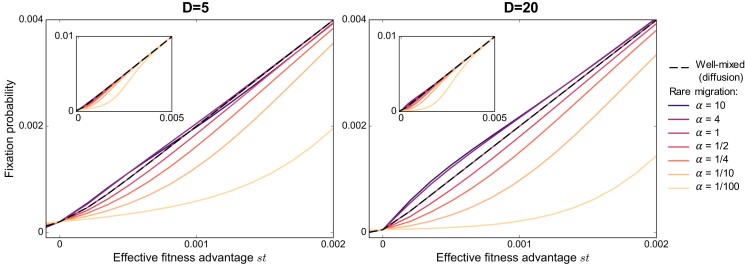
Mutant fixation probability in the star: rare migration regime. The analytical fixation probability in the rare migration regime of a single mutant in a star graph is plotted versus effective fitness advantage *st* with D=5 (left) or 20 (right) demes and K=1,000 individuals per deme on average at the bottleneck (see Supplementary Section 2.2). Mutants are initially placed uniformly at random at a bottleneck. Results are shown for various values of migration asymmetry $\alpha =m_{O}/m_{I}$ (see Fig. 1). Black dashed lines give the fixation probability obtained in the diffusion approximation for a well-mixed population with KD=5,000 individuals initialized with one mutant (see Supplementary Section 1.2). Insets show a larger range of effective fitness advantages than main panels.

We showed that no graph can amplify natural selection when migrations are frequent, using a branching process approach. How can this be reconciled with the findings of amplification in evolutionary graph theory (14)? To address this question, let us consider the rare migration regime in our model. When migrations occur on a longer timescale than the time needed for one mutant to fix in a deme, the graph can be described in a coarse-grained way as having demes that are either fully mutant or fully wild-type (34, 35). These states can be directly mapped to those of a graph with a single individual per node, as considered in evolutionary graph theory. This mapping breaks down for more frequent migrations, as demes can include various proportions of mutants and wild-types. We studied the rare migration regime in Ref. (34), and these results can easily be adapted to our serial dilution model (see Supplementary Section 2). For rare migrations, the star can amplify natural selection if α>1, where $\alpha =m_{I}/m_{O}$ quantifies the asymmetry between migrations incoming and outgoing to and from the center (see Fig. 1). Conversely, it suppresses selection when α<1. In particular, starting from a fully mutant deme and for rare migrations, our model exactly maps to evolutionary graph theory under the Birth–death update rule if α=D−1 (34). Starting from a single mutant, it first needs to fix in its deme before it may spread to other ones. Therefore, its probability of fixation is the product of that in a deme and of that starting from a fully mutant deme. In Fig. 4, we show rare migration results for the star starting from a single mutant. We observe that amplification is weak, even though it becomes larger when the number of demes increases. In addition, amplification is restricted to small fitness advantages, see also Ref. (41). Here, we observe that it exists for *st* of order 1/K. Increasing the fitness of the mutant makes the fixation probability converge to that of the well-mixed population (Fig. 4, insets).

**Fig. 5.**
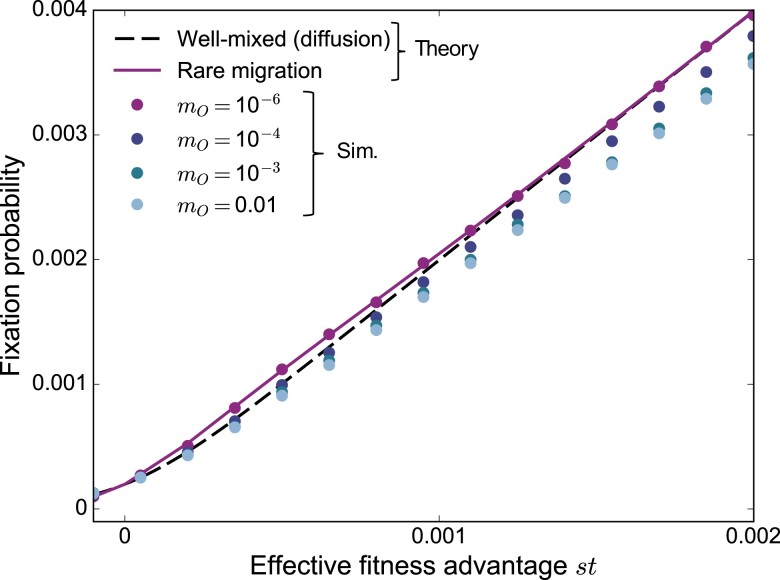
Mutant fixation probability in the star: from rare to frequent migrations. The fixation probability of a single mutant in a star graph is shown versus effective fitness advantage *st* with D=5, K=1,000 and α=4. Lines are analytical predictions, shown for rare migrations (see Supplementary Section 2.2), as well as for a well-mixed population of size KD=5,000 in the diffusion approach (see Supplementary Section 1.2). Markers are simulation results (“Sim.”) obtained over 4 million realizations for $m_{O}=10^{-6}$, and 1 million for other values of $m_{O}$. For $m_{O}=10^{-6}$, we are in the rare migration regime, but we exit it as migration probabilities increase above this value (see Supplementary Section 2).

How do our results for frequent migrations connect to those for rare migrations? Simulations allow us to bridge these two regimes. In Fig. 5, we focus on the star with α=4, which features amplification of natural selection when migrations are rare. We find that increasing migration probabilities leads to suppression, even in the regime of mutant fitness advantages where amplification exists for rare migrations. In the rare migration regime, a mutant must fix in one deme before it can spread. Since the mutant is placed in a deme chosen uniformly at random, most mutants start in a leaf. When α>1, a fully mutant leaf is more likely to send a mutant individual to the center than to receive a wild-type one. This asymmetry provides an extra advantage to a weakly beneficial mutant placed in a leaf. When migrations are frequent, fixation does not occur deme by deme, and this effect disappears.

## Discussion

We have proposed a model of spatially structured populations on graphs, where each node of the graph comprises a well-mixed deme. The population evolves through serial within-deme growth steps and dilution-migration steps. Within a branching process approach, we showed that for frequent migrations, suppression of natural selection is pervasive when there is asymmetry between the total incoming and outgoing migration flows to and from a deme, and becomes stronger when this asymmetry does. Conversely, for circulation graphs where there is no such asymmetry, spatial structure has no effect on mutant fixation probability. Our key result that spatial structure suppresses selection for frequent asymmetric migrations holds for mutants initially placed uniformly at random. Mutants starting in advantaged demes, which send more individuals to other demes than they receive from them, are more likely to fix than in a well-mixed population, while the opposite holds for other demes. However, averaging over all demes can only yield suppression of selection, due to the properties of the generating function of the branching process, from which extinction probabilities are derived. In addition to these general results, we studied in detail the star, which can amplify or suppress natural selection depending on the update rule in evolutionary graph theory (14, 18, 20, 21). With a deme on each node, the star amplifies selection for rare migrations and small fitness advantages, if incoming migrations to the center are stronger than outgoing ones (34). Here, we showed that it becomes a suppressor of selection for more frequent migrations, provided there is asymmetry between incoming and outgoing migrations to and from the center. In this regime, the star also accelerates evolutionary dynamics compared to a well-mixed population.

Our model establishes a link between classical population genetics models (9–13) and evolutionary graph theory (14, 18, 19). Indeed, our finding that circulation graphs do not affect mutant fixation probability is consistent with Maruyama’s theorem (11, 12) and with the circulation theorem in evolutionary graph theory (14), and connects them. Furthermore, in the rare migration regime, with appropriately chosen migration asymmetries, we recover results from evolutionary graph theory (34). However, we find that amplification of natural selection is limited to rare migrations and small fitness advantages, while suppression of selection is pervasive for frequent migrations, when they are asymmetric.

The impact of spatial structure on population genetics has often been discussed in terms of effective population sizes. For a given quantity, the effective size of a structured population is the size of a well-mixed population that would yield the same value of this quantity. Thus, the effective size may depend on the quantity of interest (42), and may not exist (43). Several effective sizes have been employed to characterize spatially structured populations. The inbreeding effective size is associated to the probability of identity-by-descent of two randomly chosen neutral alleles (44, 45). The coalescent effective size may be found by looking for the scaling of time to retrieve the standard coalescent (43, 46). In the diffusion approximation, mapping the per-generation mean and variance of the change in mutant frequency to those of a well-mixed population may allow to define an effective selection coefficient and a variance effective population size (47). While these effective population sizes characterize neutral evolution well, they may not suffice to describe evolution under selection (13, 45, 46). As an illustration, for Wright’s island model (9), i.e. for the clique, the variance effective size is larger than the actual one, but the effective selection coefficient is smaller than the actual one, their product being preserved, consistently with Maruyama’s result that fixation probability is unaffected (47). Thus, while at least the inbreeding effective size can be calculated within our model, following (45), it does not directly shed light on the fixation of beneficial mutants.

Our serial dilution model bridges the gap between theory and experiments. Indeed, batch culture setups with serial transfers are commonly used in evolution experiments (26–33). Experimentally, it is possible for the dilution step to incorporate exchanges between different demes, thereby allowing to investigate spatially structured populations (29–31, 33). Importantly, our results depend on migration asymmetry, which can be directly tuned in such experiments. Experiments with asymmetric migrations were recently initiated in Ref. (33), in the regime of large fitness advantage of the mutant. We hope that our work will open the way to more quantitative comparisons between theoretical predictions and experimental results for spatially structured populations.

Our branching process approach provides analytical predictions in the regime of large populations sizes, nonrare migrations and small fitness advantage of the mutant. Another important theoretical approach to study populations dynamics, which holds in a larger regime of parameters, is the diffusion approximation (48). This approach is well-established for well-mixed populations (42), and has been extended to coupled Wright–Fisher models describing several alleles on multiple loci at linkage equilibrium (49). Building upon the link with coalescent theory (50, 51), these descriptions are the subject of thorough mathematical analysis, and allow for exact simulation methods (52, 53). An interesting perspective would thus be to study our model of spatially structured population within the diffusion approximation, building upon the link with structured Wright–Fisher models (54, 55). In particular, it would allow us to study the fate of deleterious mutants. Besides, in this work, we have focused on the fate of mutants that are introduced at a bottleneck. Thus, another extension would be to consider mutants that can appear at any division during the growth phase, building on studies of growth and dilution models for well-mixed populations (56–61). Beyond the fate of a mutant, investigating how spatial population structure impacts long evolutionary trajectories in our model would be very interesting (62), as well as considering regimes where multiple mutant lineages coexist (28, 63). Another important extension would be to incorporate changing environments (64–70), and to address cases where demes can go extinct (13). Finally, the impact of spatial structure on mutant fixation is also important in expanding populations. Indeed, the expanding front features reduced effective population sizes and reduced competition. Mutants can then take over by a phenomenon known as gene surfing (71, 72). Connecting these continuous models of expanding populations to the present discrete models of populations with fixed spatial structure, and addressing population expansion in models on graphs, are interesting topics for future work.

## Methods

### Model of spatially structured populations on graphs with serial dilutions

In our serial dilution model, we consider a connected graph with *D* nodes, each comprising a well-mixed deme, and with migration probabilities $m_{ij}$ between each pair of demes $(i,j)\in \{1,\ldots ,D{\}}^{2}$. An elementary step of the dynamics is shown in Fig. 1 and includes two phases.

The demes first undergo deterministic exponential growth for time *t*. The growth rates are $f_{W}=1$ for wild-types and $f_{M}=1+s$ for mutants. Denote by $M_{i}$ (resp. $W_{i}$) the numbers of mutants (resp. wild-types) in deme *i* at the bottleneck of interest, and by $x_{i}=M_{i}/(M_{i}+W_{i})$ the mutant fraction in deme *i* at this bottleneck. After growth, the total number of individuals is $N_{i}^{\prime }=M_{i}e^{t}+W_{i}e^{(1+s)t}$, which is very large (as long as *t* is not too small), and the fraction of mutants is $x_{i}^{\prime }=x_{i}e^{st}/[1+x_{i}(e^{st}-1)]$.

Then, a dilution and migration step is carried out through independent binomial samplings. For each ordered pair of demes (i,j), including i=j, two binomial samplings (one for each type, namely mutants and wild-types) take place simultaneously. Each of the $N_{i}^{\prime }$ individuals in deme *i* can be sampled, and each type is sampled proportionally to its frequency after growth. Thus, we sample the number of mutants (resp. wild-types) that migrate from *i* to *j* from a binomial law, with $N_{i}^{\prime }$ trials, and probability of success $Km_{ij}x_{i}^{\prime }/N_{i}^{\prime }$ (resp. $Km_{ij}(1-x_{i}^{\prime })/N_{i}^{\prime }$). On average, $Km_{ij}$ individuals migrate from deme *i* to *j*, resulting into a new bottleneck comprising $K\sum_{i}m_{ij}$ individuals in deme *j*. Assuming $\sum_{i}m_{ij}=1$ for all *j*, the average bottleneck size of all demes is *K*. Selection is soft, i.e. the contributions of demes are not affected by their average fitnesses (73). Modeling all exchanges between demes through independent binomial samplings allows us to account for fluctuations that would happen at the dilution step in an experiment.

While the bottleneck size is not strictly fixed in our model, a variant where it is fixed, and where dilution and growth events are performed via multinomial sampling, yields very similar results in the regimes studied here. Note that this multinomial variant can be helpful for small deme sizes, where bottleneck size fluctuations may yield extinctions otherwise. For a single deme, such models with dilution and growth are very close to the Wright–Fisher model, with each bottleneck mapping to a generation (74, 75) (see Supplementary Section 1). Therefore, our model is close to a structured Wright–Fisher model. However, note that structured Wright–Fisher models assume binomial sampling within each deme after deterministic migration of offspring (54, 55). In Section 5 of the Supplementary Material, we present two variants of our model, one with multinomial sampling, and the other with binomial sampling within each deme after deterministic migration of offspring. We find the same results with all three variants (see in particular Supplementary Fig. S5), which demonstrates the robustness of our conclusions.

We perform stochastic simulations of this model. We also obtain analytical results, using a branching process approach, outlined below.

### Branching process analysis

We describe the state of the population using a multitype branching process approach, where each type represents each deme, and the number of mutants in each deme is followed (76, 77). The branching process description assumes that all mutant lineages are independent (76). Under this hypothesis, considering one mutant located in deme *i* at a given bottleneck, the numbers of its descendants $\left(n_{1},\ldots ,n_{D}\right)$ in the *D* demes at the next bottleneck follow a probability distribution $\phi_{i}(n_{1},\ldots ,n_{D})$. These descendants are the mutants that grew from the initial one in deme *i*, and were then sampled to any destination deme at the migration and dilution step. Assuming independent mutant lineages is valid when mutants are in small numbers, and when deme sizes are all large, i.e. K≫1. It holds at early phases starting from a single mutant, but fails if the number of mutants becomes large. For mutants with substantial selective advantage, namely for st≫1/K here, extinction events happen when mutants are still rare, due to stochastic fluctuations associated to sampling. Indeed, in a well-mixed population, if their fraction reaches a given threshold, beneficial mutants are very likely to fix in the end (78, 79). Therefore, the branching process approach yields accurate results on extinction probabilities and extinction times provided that K≫1 and Kst≫1.

Starting from one single mutant in deme *i*, the probability $\phi_{i}(n_{1},\ldots ,n_{D})$ to have $\left(n_{1},\ldots ,n_{D}\right)$ mutants at the next bottleneck is given by the growth and migration-dilution process described above, where mutants migrating to different demes are sampled independently from binomial distributions. We then define a multidimensional generating function f for $x\in [0,1{]}^{D}$, via its components


(1) $$f_{i}(x)=\sum_{n_{1},\ldots ,n_{D}=0}^{\infty }\phi_{i}(n_{1},\ldots ,n_{D})\prod_{j=1}^{D}x_{j}^{n_{j}}\;\text{ for }i=1,\ldots ,D.$$

Let $p_{i}$ denote the mutant extinction probability starting from one mutant in deme *i*. The vector of extinction probabilities $p=(p_{1},\ldots ,p_{D})$ is the only fixed point of f that is not equal to (1,…,1). To solve the fixed point equation p=f(p), we assume st≪1 (jointly with K≫1 and Kst≫1) and write a Taylor expansion of this equation in *st*. This allows to determine the extinction probabilities $p_{i}$ in this regime, and the fixation probabilities $\rho_{i}=1-p_{i}$.

In the regime of frequent migrations, where all nonzero migration probabilities are much larger than *st*, the binomial distributions used in sampling can be approximated by Poisson ones, see Supplementary Section 3. For rarer migrations, the full binomial distributions have to be used, and the Taylor expansions need to account for how migration probabilities scale in *st*, see Supplementary Section 4. In the Supplementary material, we explicitly address the case of frequent migrations, as well as the cases where all exchanges between different demes are of order *st* or $(st{)}^{2}$.

Using iterates of the generating function, we can also derive the probability for mutants to be extinct at a given bottleneck, and then the average time to extinction (see Supplementary Section 3.1.1).

## Supplementary Material

pgad392_Supplementary_Data

## Data Availability

All relevant data is included in the main text or in the supplementary material. Python code for our numerical simulations, allowing to reproduce the figures, is freely available at https://github.com/Bitbol-Lab/Structured_pop.

## Preprints

This manuscript was posted as a preprint with https://www.biorxiv.org/content/10.1101/2023.06.19.545559.
