# Supplementary material for: Frequent asymmetric migrations suppress natural selection in spatially structured populations
Source: PNAS Nexus. 2023 Nov 14;2(11):pgad392. doi: 10.1093/pnasnexus/pgad392 (PMC10667037; doi:10.1093/pnasnexus/pgad392)
Supplement: pgad392_Supplementary_Data [file pgad392_supplementary_data.pdf]

Supplementary Material for "Frequent asymmetric migrations suppress natural selection in spatially structured populations"

Alia Abbara[1,2], Anne-Florence Bitbol[1,2,*]

1 Institute of Bioengineering, School of Life Sciences, École Polytechnique Fédérale de Lausanne (EPFL), CH-1015 Lausanne, Switzerland
2 SIB Swiss Institute of Bioinformatics, CH-1015 Lausanne, Switzerland
* Corresponding authors: alia.abbara@epfl.ch, anne-florence.bitbol@epfl.ch

November 8, 2023

Contents

1 Serial dilution model for a well-mixed population **2**
1.1 Model . . . 2
1.2 Diffusion approximation . . . 3
1.3 Branching process approximation . . . 3

2 Rare migration regime for structured populations on graphs **4**
2.1 Definition of the rare migration regime . . . 4
2.2 Fixation probability for the star graph . . . 5

3 Structured populations on graphs with serial dilutions: branching process with Poisson sampling **5**
3.1 When all demes start at K on average . . . 5
3.1.1 General description . . . 5
3.1.2 Spatial structure cannot amplify selection within the branching process regime . 7
3.1.3 Expansion of the extinction probability . . . 8
3.2 When all demes contribute by K on average . . . 9
3.3 Strongly symmetric graphs . . . 10
3.3.1 Some circulation graphs: clique and cycle . . . 10
3.3.2 The star graph . . . 10
3.4 Circulation theorem: all circulation graphs have the same fixation probability . . . 12
3.4.1 Connected circulation graphs are strongly connected . . . 12
3.4.2 Property of the migration matrix of a circulation graph . . . 13
3.4.3 Proof of the circulation theorem . . . 13
3.4.4 Circulation graphs have the same fixation probability as the well-mixed population 15
3.5 Circulations have the same average extinction time as a well-mixed population . . . 16
3.6 All graphs that are not circulations strictly suppress selection . . . 16
3.6.1 When all demes start at K on average . . . 17
3.6.2 When all demes contribute by K on average . . . 17

4 Structured populations on graphs with serial dilutions: branching process with binomial sampling **18**
4.1 Motivation . . . 18
4.2 When all demes start at K on average . . . 19
4.2.1 Generating function . . . 19
4.2.2 Extinction probabilities: general expansion . . . 19
4.2.3 Well-mixed population . . . 20

4.2.4 When all migration probabilities are of order 1 . . . 20
4.2.5 When exchanges between different demes are of order st . . . 20
4.2.6 When exchanges between different demes are of order $(st)^2$ . . . 21
4.2.7 Application to the star graph . . . 21
4.2.8 Numerical results on fixation dynamics . . . 22
4.3 When all demes contribute by K on average . . . 23

5 Different models for serial dilution in structured populations **25**

6 Dirichlet cliques **26**
6.1 Definition of the Dirichlet distribution . . . 26
6.2 Examples of Dirichlet distributions . . . 27
6.3 Application: Dirichlet cliques . . . 28

1 Serial dilution model for a well-mixed population

Our goal is to develop a serial dilution model for spatially structured populations on graphs. Let us first present the serial dilution model in the simple case of a well-mixed population. We discuss the mutant fixation probability and average extinction time in this model. We do so first within the diffusion approximation (see also [1]), and next within the coarser branching process approximation.

1.1 Model

Hypotheses. Consider a model with serial dilutions for a well-mixed population of initial bottleneck size K. First, it undergoes deterministic exponential growth for a time t. Then, K individuals are selected randomly from the grown population to form the next "generation", and so on. We assume that there are two types of individuals, wild-types with fitness 1 and mutants with fitness $1 + s$. These fitnesses represent deterministic growth rates in the exponential phase. We consider that the sampling is done with replacement from the grown population, and follows a binomial law, as in the Wright-Fisher model.

Variation of the mutant fraction. Assume that at generation n the initial fraction of mutants (among the K founding individuals of this generation) is x_n. After growth, the fraction of mutants reads

$$x'_n = \frac{x_n e^{st}}{1 + x_n(e^{st} - 1)} . \tag{S1}$$

Introducing

$$\sigma = e^{st} - 1 , \tag{S2}$$

we get

$$x'_n = \frac{(1+\sigma)x_n}{1 + \sigma x_n} . \tag{S3}$$

As K individuals are sampled using a binomial law with proportion x'_n, the mean shift of the mutant fraction from generation n to generation $n + 1$ is

$$M(x_n) \equiv \langle \delta x_n \rangle = \langle x_{n+1} \rangle - x_n = x'_n - x_n = \frac{\sigma x_n(1 - x_n)}{1 + \sigma x_n} \approx \sigma x_n(1 - x_n) , \tag{S4}$$

where the mean denoted by $\langle . \rangle$ is over the possible values of $\delta x_n = x_{n+1} - x_n$ at a given x_n, and where we have assumed $|\sigma| \ll 1$ in the last expression. In addition, the variance of the fraction x_{n+1} of mutant organisms in generation $n + 1$ reads

$$\Delta x^2_{n+1} = \frac{x'_n(1 - x'_n)}{K} . \tag{S5}$$

This yields

$$V(x_n) \equiv \langle (\delta x_n)^2 \rangle - \langle \delta x_n \rangle^2 = \Delta x^2_{n+1} = \frac{(1+\sigma)x_n(1 - x_n)}{K(1 + \sigma x_n)^2} \approx \frac{x_n(1 - x_n)}{K} , \tag{S6}$$

where we have assumed $|\sigma| \ll 1$ again.

1.2 Diffusion approximation

Mutant fixation probability. Eqs. S4 and S6 exactly map to their Wright-Fisher equivalents, with σ standing for the fitness advantage of the mutant and K standing for the fixed population size in the Wright-Fisher model. Thus [2], within the diffusion approximation, which assumes $K \gg 1$, the fixation probability $\rho(x)$ of the mutant type starting from a fraction x of mutants satisfies the steady-state Kolmogorov backward equation

$$\frac{V(x)}{2}\frac{d^2\rho}{dx^2} + M(x)\frac{d\rho}{dx} = 0\,, \tag{S7}$$

where $M(x)$ and $V(x)$ are given above. In addition, $\rho(0) = 0$ and $\rho(1) = 1$. Therefore, for $K \gg 1$ and $|\sigma| \ll 1$, we have

$$\rho(x) = \frac{1 - e^{-2K\sigma x}}{1 - e^{-2K\sigma}}\,. \tag{S8}$$

If $K \gg 1$ and $|s|t \ll 1$, combining Eq. S2 and Eq. S8 yields

$$\rho(x) = \frac{1 - e^{-2Kstx}}{1 - e^{-2Kst}}\,. \tag{S9}$$

This exactly matches the Wright-Fisher result, but with st replacing the fitness advantage of the mutant and K standing for the fixed population size in the Wright-Fisher model. In fact, for $t = 1$ we recover the Wright-Fisher result with population size $N = K$. In particular, starting from one mutant at the bottleneck, $x = 1/K$, we have

$$\rho(1/K) = \frac{1 - e^{-2st}}{1 - e^{-2Kst}}\,. \tag{S10}$$

Fixation and extinction times. The average fixation time or extinction time (absorption conditioned on fixation or extinction) starting from an initial frequency x of mutants can also be calculated in the diffusion approximation for the Wright-Fisher process [3]. This can be adapted to our serial dilution model.

1.3 Branching process approximation

The branching process approximation is coarser than the diffusion approximation, but it will be extremely useful to study our spatially structured populations on graphs. Therefore, here, we present it for the serial dilution model for a well-mixed population.

Hypotheses. Consider a well-mixed population of bottleneck size K in our serial dilution model, starting from the state where there is 1 mutant and $K-1$ wild-type organisms. In our model, the number of mutants sampled to make generation $n+1$ follows a binomial distribution with parameters K and x'_n (see Eq. S3). If $K \gg 1$, $x'_n \ll 1$ and Kx'_n is of order 1, then this binomial distribution is well approximated by a Poisson distribution with mean Kx'_n. In particular, at the first generation, with $x_0 = 1/K \ll 1$, the relevant Poisson distribution has mean

$$\lambda = K\frac{(1+\sigma)x_0}{1+\sigma x_0} = \frac{1+\sigma}{1+\sigma/K} = 1 + \sigma - \frac{\sigma}{K} + O\left(\frac{\sigma^2}{K^2}\right). \tag{S11}$$

Let us assume in addition that $|s|t \ll 1$ and $K|s|t \gg 1$: then, $\sigma = st + (st)^2/2 + o((st)^2)$, and $|s|t/K \ll (st)^2$, yielding

$$\lambda = 1 + st + \frac{(st)^2}{2} + o\left((st)^2\right)\,. \tag{S12}$$

Assuming that different mutant lineages can be considered independent, which is acceptable if the population is large enough and the mutant fraction remains small enough while the fate of the mutant is set, the number of mutants in the population follows a branching process with offspring distribution given by the Poisson law with mean λ [4]. The associated generating function is $g : x \mapsto \exp\left[\lambda\,(x-1)\right]$.

Mutant fixation probability. The probability p of extinction of the mutant can be calculated using a branching process approach, as for the Wright-Fisher process [5]. Indeed, the extinction probability p is a fixed point of the generating function [4], i.e. it satisfies

$$p = g(p) = \exp\left[\lambda\left(p-1\right)\right] . \tag{S13}$$

If $\lambda \leq 1$, corresponding to $s \leq 0$ (see Eq. S12), the only solution of Eq. S13 is $p = 1$, and extinction is certain. Note that this illustrates that this approach is not taking genetic drift into account properly, and instead focuses on $K \gg 1$. Let us thus focus on the regime where $s > 0$, $st \ll 1$, $K \gg 1$ and $Kst \gg 1$. We then expect the extinction probability p to be close to 1. Let us expand it in powers of st:

$$p = 1 - ast + \frac{b}{2}(st)^2 - \frac{c}{6}(st)^3 + o((st)^3) . \tag{S14}$$

To determine a and b, let us use Eq. S13, injecting the expansions of λ and p from Eqs. S12 and S14:

$$\begin{aligned}
&1 - ast + \frac{b}{2}(st)^2 - \frac{c}{6}(st)^3 + o((st)^3) \\
&\quad = \exp\left[\left(1 + st + \frac{(st)^2}{2} + o((st)^2)\right)\left(-ast + \frac{b}{2}(st)^2 - \frac{c}{6}(st)^3 + o((st)^3)\right)\right] \\
&\quad = \exp\left[-ast + \frac{1}{2}\left(b-2a\right)(st)^2 + \frac{1}{6}\left(-c+3b-3a\right)(st)^3 + o((st)^3)\right] \\
&\quad = 1 - ast + \frac{1}{2}\left(b - 2a + a^2\right)(st)^2 + \frac{1}{6}\left(-c + 3b - 3a - 3ab + 6a^2 - a^3\right)(st)^3 + o((st)^3) .
\end{aligned} \tag{S15}$$

Identifying terms in this expansion in powers of s, we obtain $a = 2$ and $b = 10/3$. Therefore, the fixation probability of the mutant is

$$\rho = 1 - p = 2st - \frac{5}{3}(st)^2 + O((st)^3) . \tag{S16}$$

To first order in st, this is consistent with Eq. S10 in the regime where $s > 0$, $st \ll 1$, $K \gg 1$ and $Kst \gg 1$. Note however that there is a slight discrepancy on the second order term, whose prefactor is -2 in the diffusion approximation and $-5/3$ here.

Average mutant extinction time. The average mutant extinction time can be obtained from the successive iterates of the generating function [4]. See Eq. S31 below for the general case of the structured population, which can be reduced to the simpler case of the well-mixed population.

2 Rare migration regime for structured populations on graphs

2.1 Definition of the rare migration regime

When exchanges between different demes are rare enough, each deme behaves like an isolated well-mixed population, and has time to become completely wild-type or completely mutant, before any migration events occurs. This defines the rare migration regime. Let $t^{(fix)}$ be the fixation time of one mutant in a well-mixed population of bottleneck size K, corresponding to that of a single deme. If m_{ij} is the migration probability between different demes i and j at each dilution step, the average number of individuals sent from i to j after T dilution steps is $Km_{ij}T$. In the rare migration regime, no individual is typically exchanged between demes before a mutant has time to reach fixation in a deme. This implies the condition

$$\begin{aligned}
Km_{ij}t^{(fix)} &\ll 1 \\
m_{ij} &\ll \frac{1}{Kt^{(fix)}} .
\end{aligned} \tag{S17}$$

The average fixation time can be calculated in the diffusion approximation for the Wright-Fisher process [3], and adapted to our serial dilution model. For example, a mutant of fitness advantage $st = 0.01$ fixes in a well-mixed population of bottleneck size $K = 1000$ in $t^{(fix)} \approx 7 \times 10^2$ dilution steps on average. Therefore, a graph of deme bottleneck size $K = 1000$ with $st = 0.01$ is in the rare migration regime when all migration probabilites between distinct demes satisfy $m_{ij} \leq 10^{-6}$.

2.2 Fixation probability for the star graph

In the rare migration regime, we use a coarse-grained description of the state of the graph via the state of the demes, which are either completely mutant or completely wild-type [6]. The dynamics of the graph is described by a Markov chain: a transition between states can happen when one migration event occurs.

Following [6], we can compute the fixation probability starting from one fully mutant deme for a star graph with D demes of size K. Let $\Phi_{(x,i)}$ be the fixation probability when the center is in state $x \in \{0, 1\}$ where 0 is wild-type and 1 is mutant, and i leaves are mutant. Then for $i = 1, ..., D-1$ we have

$$\Phi_{(0,i)} = T_{(0,i)\to(0,i-1)}\Phi_{(0,i-1)} + T_{(0,i)\to(1,i)}\Phi_{(1,i)} + [1 - T_{(0,i)\to(0,i-1)} - T_{(0,i)\to(1,i)}]\Phi_{(0,i)} \, ,$$
$$\Phi_{(1,i-1)} = T_{(1,i-1)\to(1,i)}\Phi_{(1,i)} + T_{(1,i-1)\to(0,i-1)}\Phi_{(0,i-1)} + [1 - T_{(1,i-1)\to(1,i)} - T_{(1,i-1)\to(0,i)}]\Phi_{(1,i-1)} \, ,$$

where the Ts denote transition probabilities from one state to the other upon a given migration event (see [6] for details). The above equations become in matrix form

$$\begin{pmatrix} \Phi_{(0,i)} \\ \Phi_{(1,i)} \end{pmatrix} = \begin{pmatrix} \frac{\Gamma_0 - \Gamma_1}{1+\Gamma_0} & \frac{1+\Gamma_1}{1+\Gamma_0} \\ -\Gamma_1 & 1+\Gamma_1 \end{pmatrix} \begin{pmatrix} \Phi_{(0,i-1)} \\ \Phi_{(1,i-1)} \end{pmatrix} \, , \tag{S18}$$

with $\Phi_{(0,0)} = 0$ and $\Phi_{(1,D-1)} = 1$. Here we have introduced $\Gamma_0 = \gamma/\alpha$ and $\Gamma_1 = \gamma\alpha$, with $\gamma = \rho_W/\rho_M$, where ρ_M (respectively ρ_W) is the fixation probability of the mutant starting from one mutant (respectively of the wild-type starting from one wild-type individual) in a well-mixed population of bottleneck size K. Meanwhile, $\alpha = m_I/m_O$, where m_O is the migration probability from the center to any leaf while m_I is the migration probability from any leaf to the center.

Solving Eq. S18 yields the fixation probabilities starting from a fully mutant center, or one fully mutant leaf [6]:

$$\Phi_{(1,0)} = \frac{1-\gamma^2}{1+\alpha\gamma - \gamma(\alpha+\gamma)\left(\frac{\gamma(1+\alpha\gamma)}{\alpha+\gamma}\right)^{D-1}} \, , \tag{S19}$$

$$\Phi_{(0,1)} = \frac{\alpha}{\alpha+\gamma}(1+\alpha\gamma)\Phi_{(1,0)} \, . \tag{S20}$$

The weighted average of these quantities yields the fixation probability starting from a fully mutant deme chosen uniformly at random. To obtain the fixation probabilities starting from a single mutant, one can just multiply these quantities by ρ_M.

Here we employ a serial dilution model while [6] used a model with individual birth (with logistic rate) and death. Formally, this only affects γ, in two ways. First, in [6] it also involved the ratio of the equilibrium wild-type to mutant deme sizes (which was in practice close to one), while here this ratio is one, as the bottleneck size is imposed. Second, in [6] the fixation probabilities in demes were computed within the Moran model, while here they should be computed within the serial dilution model, i.e. using Eq. S10 in the diffusion approximation. Apart from these minor differences, the rare migration regime is the same here as in [6].

3 Structured populations on graphs with serial dilutions: branching process with Poisson sampling

3.1 When all demes start at K on average

3.1.1 General description

Model. Let us now consider a graph with D nodes, and a well-mixed deme on each of these nodes. This spatially structured population undergoes successive steps of separate exponential growth in each demes and serial dilution and migration events, as described in the main text. We assume that the migration probabilities m_{ij} satisfy $\sum_{i=1}^{D} m_{ij} = 1$ for all j, ensuring that all demes start at an average bottleneck size K. We ask about the fate of a mutant that is introduced in deme i, with $i \in \{1, \dots, D\}$,

at a bottleneck, the rest of the population being fully wild-type. The initial mutant fraction in deme i is $x_0^{(i)} = 1/K$. After exponential growth, the mutant fraction in deme i is given by (see Eq. S1):

$$x_0'^{(i)} = \frac{x_0^{(i)} e^{st}}{1 + x_0^{(i)}(e^{st} - 1)} = \frac{\lambda}{K}\,, \tag{S21}$$

where λ is defined in Eq. S11. Let us assume $s > 0$, $st \ll 1$, $K \gg 1$ and $Kst \gg 1$ (as above for the branching process approximation): λ is then given by Eq. S12.

Poisson approximation. Upon the first dilution-migration step, we sample on average $Km_{ij}x_0'^{(i)}$ mutants that go to deme j using binomial sampling from the grown deme i. The relevant binomial law is $\mathcal{B}\left(N_i', Km_{ij}x_0'^{(i)}/N_i'\right)$, where N_i' is the size of deme i after exponential growth. If $N_i' \gg 1$ and $Km_{ij}x_0'^{(i)}/N_i' \ll 1$ while $\lambda_{ij} = Km_{ij}x_0'^{(i)}$ is of order 1, we can approximate this by the Poisson distribution with mean

$$\lambda_{ij} = Km_{ij}x_0'^{(i)} = \lambda m_{ij}\,, \tag{S22}$$

where we used Eq. S21. Note that if m_{ij} is of order st or smaller, then λ_{ij} is not of order 1: in this case, the Poisson approximation is not justified. (Besides, our upcoming identification of terms in expansions in powers of st will not hold in these cases.) Therefore, let us assume that all m_{ij} that are nonzero are of order 1 (and in particular, $m_{ij} \gg st \gg 1/K$). Note that this is not compatible with the rare migration hypothesis which requires $m_{ij} < 1/K$. (Besides, the fact that the rare migration hypothesis requires considering fixation in demes is incompatible with the assumption that the fate of the mutant is set while its fraction remains small, which enters the branching process approximation.) Hence, here we consider the regime of intermediate migrations. In Section 4, we will deal with rarer migrations, of order st or $(st)^2$.

Multi-type branching process. The full process can be formally described by $\mathbf{Z}^{(0)}, \mathbf{Z}^{(1)}, \ldots$, where $\mathbf{Z}^{(k)}$ is a vector that gives the state of our graph at bottleneck step k, with the i-th component $Z_i^{(k)}$ being the number of mutants in deme i. $\mathbf{Z}^{(0)}$ is the initial state of the graph: here, $\mathbf{Z}^{(0)} = \mathbf{e}_i$, the vector with only 0s except one 1 at position i, since we initialize with a single mutant in deme i at the bottleneck. Assuming large demes and independence of all mutant lineages, we can use the formalism of multi-type branching processes [4, 7] to describe the state of our population. Each "type" in the branching process corresponds to a different deme in our system. This description generalizes the branching process approximation seen above for the well-mixed population in the serial dilution model.

Generating function. The (multivariate) generating function of our multi-type branching process is a function $\mathbf{f} : [0,1]^D \to \mathbb{R}^D$ with D components $f_i : [0,1]^D \to \mathbb{R}$ defined as follows for all $i \in \{1, \ldots, D\}$:

$$f_i : \mathbf{x} \mapsto \sum_{r_1,..,r_D=0}^{\infty} \left(\prod_{j=1}^{D} x_j^{r_j} \right) \phi_i(r_1, ..., r_D)\,, \tag{S23}$$

where $\phi_i(r_1, ..., r_D)$ is the probability that starting from one mutant located in deme i, the numbers of mutants at the next bottleneck are $r_1, ..., r_D$. In the present case, since

$$\phi_i(r_1, ..., r_D) = \prod_{j=1}^{D} \frac{\lambda_{ij}^{r_j}}{r_j!} e^{-\lambda_{ij}}\,, \tag{S24}$$

this yields

$$f_i : \mathbf{x} \mapsto \exp\left[\lambda \sum_j m_{ij}(x_j - 1)\right]. \tag{S25}$$

As for regular branching processes, the generating function of $\mathbf{Z}^{(k)}$ is the k-th iterate of $\mathbf{f}$. In other words, it is $\mathbf{f}^{(k)}$, with components $f_i^{(k)}$ defined as

$$f_i^{(k)}(\mathbf{x}) = f_i(\mathbf{f}^{(k-1)}(\mathbf{x})) \quad \forall i = 1, ..., D. \tag{S26}$$

Matrix of first moments. Let M be the matrix of first moments, defined by its elements

$$M_{ij} = \frac{\partial f_i}{\partial x_j}(1, ..., 1). \tag{S27}$$

Computing this matrix yields $M_{ij} = \lambda m_{ij}$. Recall that $\lambda = 1 + st + O((st)^2) > 1$ (as we assume $s > 0$). In Section 3.6.1, we will show that the migration matrix has 1 as largest eigenvalue. Therefore the largest eigenvalue of M is λ. This eigenvalue affects the properties of the branching process [4].

Extinction probabilities. Let p_i be the probability of extinction of the mutant, starting from one initial mutant in deme i, i.e. $\mathbf{Z}^{(0)} = \mathbf{e}_i$. Let $\mathbf{p} = (p_1, ..., p_D)$ be the vector composed of extinction probabilities starting from each deme. As the matrix of first moments M has eigenvalue $\lambda > 1$, Theorem 7.1 from [4] states that:

- $\mathbf{p}$ is a fixed point of the generating function: $\mathbf{p} = \mathbf{f}(\mathbf{p})$,
- $\forall i, \quad 0 \leq p_i < 1$.

Probability of extinction by generation n. The probability of having no mutants left *by* generation n is [4]

$$P[\mathbf{Z}^{(n)} = \mathbf{0}] = \mathbf{f}^{(n)}(\mathbf{0}). \tag{S28}$$

Besides, the probability of extinction is the limit when $n \to \infty$ of the iterated generating function:

$$\forall i, \quad \lim_{n\to\infty} f_i^{(n)}(\mathbf{0}) = p_i. \tag{S29}$$

The i-th component of $\mathbf{f}^{(n)}(\mathbf{0})$ grows monotonically towards p_i.

Average extinction time. The probability of becoming extinct *at* time n is

$$P[\mathbf{Z}^{(n)} = \mathbf{0} | \mathbf{Z}^{(n-1)} \neq \mathbf{0}] = f_i^{(n)}(\mathbf{0}) - f_i^{(n-1)}(\mathbf{0}). \tag{S30}$$

The vector of average extinction times (in numbers of generations) $\mathbf{t}^{(ex)} = (t_1^{(ex)}, ..., t_D^{(ex)})$, where $t_i^{(ex)}$ is the average extinction time when we start with one mutant in deme i, is given by

$$\mathbf{t}^{(ex)} = \mathbf{f}(\mathbf{0}) + \sum_{n=1}^{\infty} n[\mathbf{f}^{(n)}(\mathbf{0}) - \mathbf{f}^{(n-1)}(\mathbf{0})]. \tag{S31}$$

3.1.2 Spatial structure cannot amplify selection within the branching process regime

Within the branching process approach with Poisson sampling, let us show that the mutant fixation probability in a spatially structured population is never larger than in the well-mixed population. For this, let us start from Eq. S28, which gives the probability of extinction of mutants by bottleneck step k, starting from a single mutant. In a well-mixed population, this probability is $g^{(k)}(0)$, where $g(x) = \exp[\lambda(x-1)]$ is the generating function given by Eq. S13. For a structured population, the probability of extinction by bottleneck step k starting from an initial mutant randomly placed in the graph is $\sum_i f_i^{(k)}(\mathbf{0})/D$, where $f_i^{(k)}$ is the i-th component of the k-th iterate of the generating function defined in Eq. S25. Let us show by induction that for any graph, for all $k \geq 1$, $\sum_i f_i^{(k)}(\mathbf{0})/D \geq g^{(k)}(0)$.

First bottleneck. The probability of being extinct at the first bottleneck step in the well-mixed population is $g(0) = e^{-\lambda}$. For a structured population, it reads

$$\frac{1}{D}\sum_i f_i(\mathbf{0}) = \frac{1}{D}\sum_i \exp\left(-\lambda \sum_j m_{ij}\right) \geq \exp\left(-\frac{\lambda}{D}\sum_i\sum_j m_{ij}\right) = e^{-\lambda} = g(0)\,, \tag{S32}$$

where we have used the convexity of the exponential function, and $\sum_i m_{ij} = 1$ for all j. Thus, we have shown that $\sum_i f_i(\mathbf{0})/D \geq g(0)$. Therefore, the extinction probability at the first step is larger in the structured population than in the well-mixed one.

From bottleneck step k to $k+1$. Assume $g^{(k)}(0) \leq \sum_i f_i^{(k)}(\mathbf{0})/D$ for some $k \geq 1$. The probability that the mutant is extinct by bottleneck step $k+1$ in the well-mixed population is

$$g^{(k+1)}(0) = g(g^{(k)}(0)) = \exp\left\{\lambda \left[g^{(k)}(0) - 1\right]\right\}. \tag{S33}$$

For a structured population, it reads

$$\frac{1}{D}\sum_i f_i^{(k+1)}(\mathbf{0}) = \frac{1}{D}\sum_i \exp\left\{\lambda \sum_j m_{ij}\left[f_j^{(k)}(\mathbf{0}) - 1\right]\right\}, \tag{S34}$$

and using the convexity of the exponential function and then the induction hypothesis at bottleneck step k, we obtain:

$$\frac{1}{D}\sum_i \exp\left\{\lambda \sum_j m_{ij}\left[f_j^{(k)}(\mathbf{0}) - 1\right]\right\} \geq \exp\left\{\frac{\lambda}{D}\sum_j \sum_i m_{ij}\left[f_j^{(k)}(\mathbf{0}) - 1\right]\right\} \tag{S35}$$

$$\geq \exp\left\{\frac{\lambda}{D}\sum_j \left[f_j^{(k)}(\mathbf{0}) - 1\right]\right\} \tag{S36}$$

$$\geq \exp\left\{\lambda \left[g^{(k)}(0) - 1\right]\right\}. \tag{S37}$$

The induction hypothesis is thus verified at bottleneck step $k+1$. Therefore, we have shown by induction that for any graph, for all $k \geq 1$, $\sum_i f_i^{(k)}(\mathbf{0})/D \geq g^{(k)}(0)$.

Infinite time limit. As $k \to \infty$, we still have

$$\lim_{n\to\infty} \frac{1}{D}\sum_i f_i^{(k)}(\mathbf{0}) \geq \lim_{k\to\infty} g^{(k)}(0). \tag{S38}$$

Therefore the probability of extinction starting from a single mutant is always larger in a structured population than in a well-mixed population. In other words, there can be no amplification of natural selection in a structured population. This proof holds in the branching process regime and for frequent migrations.

3.1.3 Expansion of the extinction probability

Let us focus on the probability p_i that the mutant type gets extinct, starting from one single mutant in deme i at the bottleneck. The results in the previous subsection ensure that for all i, p_i satisfies:

$$p_i = \exp\left[\lambda \sum_{j=1}^{D} m_{ij}\left(p_j - 1\right)\right]. \tag{S39}$$

Under the assumptions $s > 0$, $st \ll 1$, $K \gg 1$ and $Kst \gg 1$, let us expand p_i perturbatively in powers of st:

$$p_i = 1 - a_i st + \frac{b_i}{2}(st)^2 - \frac{c_i}{6}(st)^3 + o((st)^3), \tag{S40}$$

where a_i, b_i and c_i are unknown prefactors. To determine a_i and b_i for each deme i, let us use Eq. S39,

using Eq. S40 and recalling that $\lambda = 1 + st + (st)^2/2 + o((st)^2)$:

$$
\begin{aligned}
p_i &= \exp\left[\left(1 + st + \frac{1}{2}(st)^2 + o((st)^2)\right)\sum_{j=1}^{D} m_{ij}\left(-a_j st + \frac{b_j}{2}(st)^2 - \frac{c_j}{6}(st)^3 + o((st)^3)\right)\right] \\
&= \exp\left[-st\sum_{j=1}^{D} m_{ij}a_j + (st)^2\sum_{j=1}^{D} m_{ij}\left(\frac{b_j}{2} - a_j\right) + (st)^3\sum_{j=1}^{D} m_{ij}\left(\frac{b_j}{2} - \frac{a_j}{2} - \frac{c_j}{6}\right) + o((st)^3)\right] \\
&= 1 - st\sum_{j=1}^{D} m_{ij}a_j + \frac{1}{2}\left[\sum_{j=1}^{D} m_{ij}b_j - 2\sum_{j=1}^{D} m_{ij}a_j + \left(\sum_{j=1}^{D} m_{ij}a_j\right)^2\right](st)^2 + \left[-\frac{1}{6}\left(\sum_{j=1}^{D} m_{ij}a_j\right)^3\right. \\
&\quad \left. + \sum_{j=1}^{D} m_{ij}\left(\frac{b_j}{2} - \frac{a_j}{2} - \frac{c_j}{6}\right) - \left(\sum_{j=1}^{D} m_{ij}a_j\right)\left(\sum_{j=1}^{D} m_{ij}\left(\frac{b_j}{2} - a_j\right)\right)\right](st)^3 + o((st)^3)\,. \qquad \text{(S41)}
\end{aligned}
$$

Combining Eqs. S40 and S41 and identifying terms in these two expansions in powers of st, we obtain:

$$
a_i = \sum_{j=1}^{D} m_{ij}a_j\,, \qquad \text{(S42)}
$$

$$
b_i = \sum_{j=1}^{D} m_{ij}b_j - 2\sum_{j=1}^{D} m_{ij}a_j + \left(\sum_{j=1}^{D} m_{ij}a_j\right)^2 = \sum_{j=1}^{D} m_{ij}b_j - 2a_i + a_i^2\,, \qquad \text{(S43)}
$$

$$
c_i = \sum_{j=1}^{D} m_{ij}c_j - 3b_i + 3a_ib_i - 3a_i + 3a_i^2 - 2a_i^3\,. \qquad \text{(S44)}
$$

3.2 When all demes contribute by K on average

So far, we assumed that the migration probabilities m_{ij} satisfy $\sum_{i=1}^{D} m_{ij} = 1$ for all j, ensuring that all demes start at an average bottleneck size K. This is the convention taken in the main text. However it is not the only possible choice. Let us now consider the case where $\sum_{j=1}^{D} m_{ij} = 1$ for all i, ensuring that all demes contribute by K on average.

As before, consider the initial condition where 1 mutant is present at the bottleneck in deme i, and the rest of the population is fully wild-type. The difference with the previous case is that the size of deme i at the bottleneck is not K but $N_i = K\sum_{k=1}^{D} m_{ki}$. Thus, the initial mutant fraction in deme i reads $x_0^{(i)} = 1/N_i$, and after exponential growth, the mutant fraction in deme i is given by (see Eq. S1):

$$
x_0'^{(i)} = \frac{x_0^{(i)}e^{st}}{1 + x_0^{(i)}(e^{st} - 1)} = \frac{\lambda}{K\sum_{k=1}^{D} m_{ki}} = \frac{1}{K\sum_{k=1}^{D} m_{ki}}\left[1 + st + \frac{(st)^2}{2} + o((st)^2)\right]\,, \qquad \text{(S45)}
$$

where we have again assumed $s > 0$, $st \ll 1$, $K \gg 1$ and $Kst \gg 1$.

Upon the first dilution-migration, we sample on average $Km_{ij}x_0'^{(i)}$ mutants that go to deme j, using binomial sampling from the grown deme i. The relevant binomial law is $\mathcal{B}\left(N_i', Km_{ij}x_0'^{(i)}/N_i'\right)$, where N_i' is the size of deme i after exponential growth. If $N_i' \gg 1$ and $Km_{ij}x_0'^{(i)}/N_i' \ll 1$ while $\lambda_{ij} = Km_{ij}x_0'^{(i)}$ is of order 1, we can approximate this by the Poisson distribution with mean

$$
\lambda_{ij} = Km_{ij}x_0'^{(i)} = \lambda\frac{m_{ij}}{\sum_{k=1}^{D} m_{ki}} \equiv \lambda\mu_{ij}\,, \qquad \text{(S46)}
$$

where we used Eq. S45 and introduced

$$
\mu_{ij} = \frac{m_{ij}}{\sum_{k=1}^{D} m_{ki}}\,. \qquad \text{(S47)}
$$

We observe that the situation is exactly the same as above, except that m_{ij} has been replaced by μ_{ij}. All the statements above can be extended to the present case by replacing m_{ij} by μ_{ij}. In particular, we obtain equations on the mutant extinction probability similar to Eq. S39, namely:

$$p_i = \exp\left[\lambda \sum_{j=1}^{D} \mu_{ij}\,(p_j - 1)\right] . \tag{S48}$$

Expanding in powers of st then yields Eq. S40, but with

$$a_i = \sum_{j=1}^{D} \mu_{ij} a_j \,, \tag{S49}$$

$$b_i = \sum_{j=1}^{D} \mu_{ij} b_j - 2a_i + a_i^2 \,, \tag{S50}$$

$$c_i = \sum_{j=1}^{D} \mu_{ij} c_j - 3b_i + 3a_i b_i - 3a_i + 3a_i^2 - 2a_i^3 \,. \tag{S51}$$

3.3 Strongly symmetric graphs

3.3.1 Some circulation graphs: clique and cycle

Clique. In the clique, all demes are equivalent, and thus a_i and b_i do not depend on i. Furthermore, migration probabilities are all identical for $i \neq j$: say that $m_{ij} = m$ for all $i \neq j$ and $m_{ii} = \tilde{m}$ for all i. We observe that the two cases distinguished above coincide, because $\sum_{i=1}^{D} m_{ij} = \sum_{j=1}^{D} m_{ij} = \tilde{m} + (D-1)m$. Furthermore, in both cases, $\tilde{m} + (D-1)m = 1$. In this context, Eq. S42 yields $a = a$ and Eq. S43 yields $b = b - 2a + a^2$, and thus $a = 2$, and we obtain an extinction probability $p = 1 - 2st$, which corresponds to that of the well-mixed population in our serial dilution and exponential growth model (compared to the Wright-Fisher population considered above (see Eq. S16), s is replaced by st). Thus, the clique structure has no effect on fixation probability in the regime addressed by the branching process formalism.

Cycle. In the cycle, all demes are equivalent, and thus a_i and b_i do not depend on i. Let us introduce $m_{i,i+1} = m_C$, $m_{i,i-1} = m_A$ and $m_{ii} = \tilde{m}$ for all i (with periodic boundary conditions). We have $\sum_{i=1}^{D} m_{ij} = \sum_{j=1}^{D} m_{ij} = \tilde{m} + m_A + m_C = 1$, and thus, the two cases distinguished above coincide. In this context, as for the cycle, Eq. S42 yields $a = a$ and Eq. S43 yields $b = b - 2a + a^2$, and thus $a = 2$. Similarly, Eq. S44 yields $b = 10/3$, and we obtain an extinction probability $p = 1 - 2st + 5/3(st)^2$: the cycle structure has no effect on fixation probability in the regime addressed by the branching process formalism.

3.3.2 The star graph

Star with $\sum_{i=1}^{D} m_{ij} = 1$ for all j (all demes start at K on average). In the star, all leaves are equivalent, but the center is different. Denoting by m_I migrations from a leaf to the center, by m_O those from the center to a leaf, and by $\tilde{m}_C$ or $\tilde{m}_L$ the self-migrations for the center and the leaf, respectively, $\sum_{i=1}^{D} m_{ij} = 1$ yields

$$\tilde{m}_C = 1 - (D-1)m_I \,, \tag{S52}$$

$$\tilde{m}_L = 1 - m_O \,. \tag{S53}$$

Eq. S42 becomes, considering the center and a leaf:

$$a_C = \tilde{m}_C a_C + (D-1)m_O a_L = \left[1 - (D-1)m_I\right] a_C + (D-1)m_O a_L \,, \tag{S54}$$

$$a_L = \tilde{m}_L a_L + m_I a_C = \left[1 - m_O\right] a_L + m_I a_C \,, \tag{S55}$$

which yields

$$a_L = \frac{m_I}{m_O} a_C = \alpha\, a_C \,, \tag{S56}$$

where we introduced the migration asymmetry $\alpha = m_I/m_O$. Then, Eq. S43 becomes, considering the center and a leaf:

$$b_C = [1-(D-1)m_I]\, b_C + (D-1)m_O b_L - 2a_C + a_C^2 \,, \tag{S57}$$

$$b_L = [1-m_O]\, b_L + m_I b_C - 2\alpha\, a_C + \alpha^2 a_C^2 \,, \tag{S58}$$

which finally yields, assuming $a_C \neq 0$,

$$a_C = 2\,\frac{1+(D-1)\alpha}{1+(D-1)\alpha^2} \quad \text{and} \quad a_L = \alpha\, a_C \,. \tag{S59}$$

Thus, the mutant fixation probabilities starting from one mutant at the bottleneck in the center or in the leaf of a star satisfying $\sum_{i=1}^{D} m_{ij} = 1$ for all j are respectively:

$$\rho_C = 2st\frac{1+(D-1)\alpha}{1+(D-1)\alpha^2} \quad \text{and} \quad \rho_L = 2st\,\alpha\frac{1+(D-1)\alpha}{1+(D-1)\alpha^2} \,. \tag{S60}$$

We note that if $\alpha = 1$, then $\rho_C = \rho_L = 2st$, indicating no impact of structure on the fixation probability in this case. More generally, migration asymmetry α has a strong impact on these probabilities. Note however that only α enters our result, not m_I and m_O separately – within the parameter regime where the branching process formalism holds. In the case where mutants are placed uniformly at random in any deme, the fixation probability is

$$\rho_a = \frac{1}{D}\rho_C + \frac{D-1}{D}\rho_L = 2st\frac{1}{D}\frac{[1+(D-1)\alpha]^2}{1+(D-1)\alpha^2} \,. \tag{S61}$$

Star with $\sum_{j=1}^{D} m_{ij} = 1$ for all i (all demes contribute by K on average). Using the same notations as above, $\sum_{j=1}^{D} m_{ij} = 1$ yields

$$\tilde{m}_C = 1-(D-1)m_O \,, \tag{S62}$$

$$\tilde{m}_L = 1-m_I \,. \tag{S63}$$

Eq. S42 becomes, considering the center and a leaf:

$$a_C = \frac{[1-(D-1)m_O]\, a_C + (D-1)m_O a_L}{1+(D-1)(m_I-m_O)} \,, \tag{S64}$$

$$a_L = \frac{[1-m_I]\, a_L + m_I a_C}{1+m_O-m_I} \,, \tag{S65}$$

which yields

$$a_L = \alpha\, a_C \,, \tag{S66}$$

exactly as above (recall that $\alpha = m_I/m_O$). Then, Eq. S43 becomes, considering the center and a leaf:

$$b_C = \frac{[1-(D-1)m_O]\, b_C + (D-1)m_O b_L}{1+(D-1)(m_I-m_O)} + a_C(a_C-2) \,, \tag{S67}$$

$$b_L = \frac{[1-m_I]\, b_L + m_I b_C}{1+m_O-m_I} + \alpha\, a_C(\alpha\, a_C - 2) \,, \tag{S68}$$

which finally yields, assuming $a_C \neq 0$,

$$a_C = 2\,\frac{m_I - m_O + \frac{1}{D-1} + \alpha\,(1+m_O-m_I)}{m_I - m_O + \frac{1}{D-1} + \alpha^2\,(1+m_O-m_I)} \quad \text{and} \quad a_L = \alpha\, a_C \,. \tag{S69}$$

Thus, the mutant fixation probabilities starting from one mutant at the bottleneck in the center or in the leaf of a star satisfying $\sum_{j=1}^{D} m_{ij} = 1$ for all j are respectively:

$$\rho_C = 2st\,\frac{m_I - m_O + \frac{1}{D-1} + \alpha\,(1+m_O-m_I)}{m_I - m_O + \frac{1}{D-1} + \alpha^2\,(1+m_O-m_I)} \quad \text{and} \quad \rho_L = \alpha\, \rho_C \,. \tag{S70}$$

We note again that if $\alpha = 1$, then $\rho_C = \rho_L = 2st$, indicating no impact of structure on the fixation probability in this case. More generally, migration asymmetry α enters these probabilities, but contrary to the previous case where migrations only entered through α, here, m_I and m_O also matter separately.

3.4 Circulation theorem: all circulation graphs have the same fixation probability

Here, we extend the circulation theorem from Ref. [8] to our model, within the branching process approximation. Consider a metapopulation on a directed graph G with a set of vertices $\mathbf{V}$, equipped with migration probabilities $m_{ij} \geq 0$ from vertex i to vertex j. G is a circulation if and only if for all i,

$$\sum_{j\in\mathbf{V}} m_{ij} = \sum_{j\in\mathbf{V}} m_{ji}\,, \tag{S71}$$

which means that the sum of outgoing migrations from i is equal to the sum of incoming migrations to i. We will restrict to connected graphs, such that there exists a path that connects each pair of vertices. In the regime of validity of the branching process approach, we aim to show that starting with a single mutant, all (connected) circulation graphs have the same probability of mutant extinction. Furthermore, this probability does not depend on the deme where the mutant started.

3.4.1 Connected circulation graphs are strongly connected

We start by showing that (connected) circulation graphs are strongly connected. A graph is strongly connected if each node can be reached from every other node. Note that this is stronger than connected because there needs to be a path from any node to any other in both directions.

Property of circulation graphs [8]. Consider a finite (connected) circulation graph G with a set of nodes $\mathbf{V}$. Let $\mathbf{A}$ be a subset of nodes from a connected component of the graph. Then

$$\sum_{i\in\mathbf{A},\, j\in\mathbf{V}} m_{ij} = \sum_{i\in\mathbf{A},\, j\in\mathbf{V}} m_{ji}\,,\ \text{i.e. ,} \tag{S72}$$

$$\sum_{i\in\mathbf{A},\, j\in\mathbf{V}\setminus\mathbf{A}} m_{ij} + \sum_{i\in\mathbf{A},\, j\in\mathbf{A}} m_{ij} = \sum_{i\in\mathbf{A},\, j\in\mathbf{V}\setminus\mathbf{A}} m_{ji} + \sum_{i\in\mathbf{A},\, j\in\mathbf{A}} m_{ji}\,, \tag{S73}$$

which results in

$$\sum_{i\in\mathbf{A},\, j\in\mathbf{V}\setminus\mathbf{A}} m_{ij} = \sum_{i\in\mathbf{A},\, j\in\mathbf{V}\setminus\mathbf{A}} m_{ji}\,. \tag{S74}$$

In other words, the sum of incoming migrations to $\mathbf{A}$ is equal to the sum of outgoing migrations from $\mathbf{A}$.

A transitive relation between strongly connected components. We define a transitive relation between two strongly components $\mathbf{A}, \mathbf{B}$. We say $\mathbf{A}$ is *positively connected* to $\mathbf{B}$ and denote $\mathbf{A} \to_+ \mathbf{B}$ if

$$\sum_{i\in\mathbf{A}, j\in\mathbf{B}} m_{ij} > 0\,.$$

Given $m > 1$ strongly connected components $\{\mathbf{A}_1, ..., \mathbf{A}_m\}$, we say that they form a *positive chain* if $\mathbf{A}_k \to_+ \mathbf{A}_{k+1}$ for $k \in \{1, ..., m-1\}$.

Proof that G is strongly connected. G can be decomposed into an ensemble $\mathcal{S}$ of $n \geq 1$ maximal strongly connected components. If $n = 1$, then $\mathcal{S} = G$ and the graph is strongly connected. Assume $n \geq 2$, let $\mathbf{A}_1 \in \mathcal{S}$. We aim to build a positive chain with distinct components of maximal length starting from $\mathbf{A}_1$.

- If $\sum_{i\in\mathbf{A}_1,\, j\in\mathbf{V}\setminus\mathbf{A}_1} m_{ij} = 0$, then $m_{ij} = 0$ for all $i \in \mathbf{A}_1$, $j \in \mathbf{V} \setminus \mathbf{A}_1$, because $m_{ij} \geq 0$. Eq. S74 then further implies that $m_{ji} = 0$ for all $i \in \mathbf{A}_1$, $j \in \mathbf{V} \setminus \mathbf{A}_1$. Thus, $\mathbf{A}_1$ is not connected to any other part of the graph, which is impossible, since the graph is connected. Therefore there exists another strongly connected component $\mathbf{A}_2 \in \mathcal{S}$ such that $\mathbf{A}_1 \to_+ \mathbf{A}_2$.
- For any $m \geq 2$, consider the set $\{\mathbf{A}_1, \mathbf{A}_2, ..., \mathbf{A}_m\} \in \mathcal{S}^m$ that completes $\{\mathbf{A}_1, \mathbf{A}_2\}$ into a positive chain of m distinct connecting components. The same argument as above implies that there exists $\mathbf{A}_{m+1} \in \mathcal{S}$, $\mathbf{A}_{m+1} \neq \mathbf{A}_m$ such that $\mathbf{A}_m \to_+ \mathbf{A}_{m+1}$. Moreover, $\mathbf{A}_{m+1} \neq \mathbf{A}_k$, $\forall k \in \{1, ..., m\}$, otherwise $(\mathbf{A}_k, \mathbf{A}_{k+1}, ..., \mathbf{A}_m, \mathbf{A}_{m+1})$ would form one larger connected component, which is impossible since the $\mathbf{A}_i$ are maximal.

- By induction we build a positive chain of distinct maximal connected components of size n, where we recall that n is the total number of maximal connected components in G. Then $\mathbf{A}_n$ should be positively connected to another element from $\mathcal{S}$, which is not in the chain. We reach a contradiction.

In conclusion, $n = 1$, and thus $\mathcal{S} = G$ and G is strongly connected.

3.4.2 Property of the migration matrix of a circulation graph

Let M be the matrix with elements $\mu_{ij} = m_{ij}/\sum_k m_{ki}$. In the convention where $\forall i$, $\sum_j m_{ji} = 1$, we have $\mu_{ij} = m_{ij}$. Furthermore, circulation graphs satisfy $\sum_k m_{ki} = \sum_k m_{ik}$, and thus, both in the convention where $\forall i$, $\sum_j m_{ji} = 1$, and in the convention where $\forall i$, $\sum_j m_{ij} = 1$, we have $\sum_k m_{ki} = \sum_k m_{ik} = 1$. This yields $\mu_{ij} = m_{ij}$ also in the convention where $\forall i$, $\sum_j m_{ij} = 1$. Therefore, in both conventions, M is the migration matrix, and all its lines and columns sum to 1. M is also non-negative. In addition, as shown above, a circulation graph is strongly connected (i.e. there is a path in each direction between each pair of vertices of the graph), which entails that M is irreducible (i.e. not similar via a permutation matrix to a block upper triangular matrix with more than one block).

The Perron-Frobenius theorem for non-negative and irreducible matrices implies that M's largest real eigenvalue ρ is positive and satisfies

$$\min_i \sum_j m_{ij} \le \rho \le \max_i \sum_j m_{ij} \,, \tag{S75}$$

and since both sums are equal to 1, $\rho = 1$. The theorem also states that ρ is a simple eigenvalue. Let $\mathcal{E}$ be the corresponding eigenspace of dimension 1. Since $\mathbb{1} = (1, ..., 1)$ is an eigenvector associated to 1, it generates $\mathcal{E}$.

3.4.3 Proof of the circulation theorem

Goal of the computation. We are interested in the expansion of p_i, the probability of extinction starting from deme i, perturbatively in powers of st that we rename x:

$$p_i = 1 + \sum_{k=1}^{\infty} \alpha_k^{(i)} x^k \,. \tag{S76}$$

Our goal is to show two points:

- For each order $k \ge 1$ and for all $i = 1, ..., D$, $\alpha_k^{(i)} = \alpha_k$, i.e. that the vector $(\alpha_k^{(i)})_{i=1,...,D} \in \mathcal{E}$. This entails that for circulations, p_i does not depend on i: a mutant has the same probability of fixation, whatever the deme i where it started.
- The equations satisfied by α_k for each $k \ge 1$ are the same for all circulations, i.e. they do not depend on the specific structure of the matrix M, provided that $\sum_k m_{ki} = \sum_k m_{ik} = 1$ for all i. This entails that the fixation probability is the same for all circulations, which extends the circulation theorem [8] to our model in the regime of the branching process approximation.

Equation on p_i. We first expand Eq. S21 as

$$\lambda = 1 + \sum_{k=1}^{\infty} \lambda_k x^k \,, \tag{S77}$$

where $\lambda_1 = 1$ (see above). We then write Eq. S39 (or, equivalently here, Eq. S48) using expansions in x:

$$p_i = \exp\left[\left(1 + \sum_{l=1}^{\infty} \lambda_l x^l\right) \sum_{j=1}^{D} m_{ij} \left(\sum_{k=1}^{\infty} \alpha_k^{(j)} x^k\right)\right] \tag{S78}$$

$$= \exp\left[\sum_{k=1}^{\infty} \beta_k^{(i)} x^k\right] \,, \tag{S79}$$

where we introduced, for $k \geq 1$:

$$\beta_k^{(i)} \equiv \sum_{j=1}^{D} m_{ij}\alpha_k^{(j)} + \sum_{l=1}^{k-1}\sum_{j=1}^{D} m_{ij}\lambda_l \alpha_{k-l}^{(j)} \,. \tag{S80}$$

Expanding the exponential and replacing p_i by its expression in Eq. S76 yields

$$1 + \sum_{k=1}^{\infty} \alpha_k^{(i)} x^k = 1 + \sum_{n=1}^{\infty} \frac{1}{n!} \left(\sum_{k=1}^{\infty} \beta_k^{(i)} x^k \right)^n \,. \tag{S81}$$

Matching each order yields for $k \geq 1$:

$$\alpha_k^{(i)} = \sum_{n=1}^{k} \frac{1}{n!} \sum_{\substack{k_1,\dots,k_n \geq 1 \\ k_1+\dots+k_n=k}} \beta_{k_1}^{(i)} \dots \beta_{k_n}^{(i)} \,. \tag{S82}$$

Induction on the order.

- **Order 1:**

At order 1, Eq. S82 reduces to

$$\alpha_1^{(i)} = \sum_{j=1}^{D} m_{ij}\alpha_1^{(j)} \,. \tag{S83}$$

In other words, the vector $(\alpha_1^{(1)}, \dots, \alpha_1^{(D)})$ is an eigenvector of M with eigenvalue 1. Thus, all its components are equal to a constant α_1.
At order 2, using $\lambda_1 = 1$, Eq. S82 reads for $i = 1, \dots, D$:

$$\alpha_2^{(i)} = \sum_{j=1}^{D} m_{ij}\alpha_2^{(j)} + \alpha_1 + \frac{1}{2}\alpha_1^2 \,. \tag{S84}$$

In vectorial terms, defining $A_2 = (\alpha_2^{(1)}, \dots, \alpha_2^{(D)})$, this equation reads

$$A_2 = MA_2 + \left(\alpha_1 + \frac{1}{2}\alpha_1^2\right) \mathbb{1} \,. \tag{S85}$$

Using the Jordan decomposition, we complete $\mathcal{E}$ into a complex basis in which M has a Jordan normal form. The complementary space of $\mathcal{E}$, that we call $\mathcal{E}^{\perp}$, is thus stable under the action of the matrix M. We decompose A_2 on this basis as $A_2 = A_{\parallel} + A_{\perp}$, with $A_{\parallel}$ in $\mathcal{E}$ and $A_{\perp}$ in $\mathcal{E}_{\perp}$. Then

$$A_{\parallel} + A_{\perp} = MA_{\parallel} + MA_{\perp} + \left(\alpha_1 + \frac{1}{2}\alpha_1^2\right) \mathbb{1} \,, \tag{S86}$$

yielding, as $A_{\parallel} = MA_{\parallel}$,

$$A_{\perp} - MA_{\perp} = \left(\alpha_1 + \frac{1}{2}\alpha_1^2\right) \mathbb{1} \,. \tag{S87}$$

Therefore, $A_{\perp} - MA_{\perp} \in \mathcal{E} \cap \mathcal{E}_{\perp} = \{0\}$, which entails $A_{\perp} \in \mathcal{E} \cap \mathcal{E}_{\perp} = \{0\}$. This determines the value of α_1 through

$$2\alpha_1 + \alpha_1^2 = 0 \,, \tag{S88}$$

which yields $\alpha_1 = -2$ for all circulations, consistent with our results obtained for the clique and the cycle above.

- **From order $k-1$ to order k:**

We assume that there exists $k \geq 2$, such that for all $l \leq k-1$, $(\alpha_l^{(1)}, ..., \alpha_l^{(D)}) = \alpha_l \mathbb{1} \in \mathcal{E}$, which directly entails $(\beta_l^{(1)}, ..., \beta_l^{(D)}) \in \mathcal{E}$. We also assume that the values of α_l for $l \leq k-1$ are the same for all circulations.

From Eq. S82 written for order k and for all $i = 1, ..., D$, we detail

$$\alpha_k^{(i)} = \beta_k^{(i)} + \sum_{n=2}^{k} \frac{1}{n!} \sum_{\substack{k_1,...,k_n \geq 1 \\ k_1+...+k_n=k}} \beta_{k_1}^{(i)} ... \beta_{k_n}^{(i)} \tag{S89}$$

$$= \sum_{j=1}^{D} m_{ij} \alpha_k^{(j)} + \sum_{l=1}^{k-1} \sum_{j=1}^{D} m_{ij} \lambda_l \alpha_{k-l}^{(j)} + \sum_{n=2}^{k} \frac{1}{n!} \sum_{\substack{k_1,...,k_n \geq 1 \\ k_1+...+k_n=k}} \beta_{k_1}^{(i)} ... \beta_{k_n}^{(i)} . \tag{S90}$$

In the last term, consider an ensemble of n integers $k_1, ..., k_n \geq 1$, such that $k_1 + ... + k_n = k$. Since $n \geq 2$, we necessarily have $k_1, ..., k_n \leq k-1$. According to the induction hypothesis, none of the $\beta_l^{(i)}$ in this sum depends on the index (i). The second term also contains $(\alpha_l^{(j)})$s with $l \leq k-1$, which thus do not depend on the index (j). We can rewrite this equation for $i = 1, ..., D$ as

$$\alpha_k^{(i)} = \sum_{j=1}^{D} m_{ij} \alpha_k^{(j)} + \gamma_k^{(i)} , \tag{S91}$$

where we introduced

$$\begin{aligned}
\gamma_k &= \sum_{l=1}^{k-1} \lambda_l \sum_{j=1}^{D} m_{ij} \alpha_{k-l}^{(j)} + \sum_{n=2}^{k} \frac{1}{n!} \sum_{\substack{k_1,...,k_n \geq 1 \\ k_1+...+k_n=k}} \beta_{k_1}^{(i)} ... \beta_{k_n}^{(i)} \\
&= \sum_{l=1}^{k-1} \lambda_l \alpha_{k-l} \sum_{j=1}^{D} m_{ij} + \sum_{n=2}^{k} \frac{1}{n!} \sum_{\substack{k_1,...,k_n \geq 1 \\ k_1+...+k_n=k}} \beta_{k_1} ... \beta_{k_n} \\
&= \sum_{l=1}^{k-1} \lambda_l \alpha_{k-l} + \sum_{n=2}^{k} \frac{1}{n!} \sum_{\substack{k_1,...,k_n \geq 1 \\ k_1+...+k_n=k}} \beta_{k_1} ... \beta_{k_n} ,
\end{aligned} \tag{S92}$$

where we employed $\sum_{j=1}^{D} m_{ij} = 1$, which holds for circulations in both conventions. Importantly, γ_k only involves lower-order terms and does not depend on i. In vectorial form, defining $A_k = (\alpha_k^{(1)}, ..., \alpha_k^{(D)})$, Eq. S91 reads

$$A_k = M A_k + \gamma_k \mathbb{1} . \tag{S93}$$

We apply the same argument as above for order 2, using the Jordan decomposition of M into a basis $\mathcal{E} \bigoplus \mathcal{E}^{\perp}$. It entails that $A_k \in \mathcal{E}$, i.e. all its components $\alpha_k^{(1)}, ..., \alpha_k^{(D)}$ are equal to a constant α_k, and $\gamma_k = 0$. Considering Eq. S91 at order $k+1$ yields $\gamma_{k+1} = 0$, i.e.

$$\sum_{l=1}^{k} \lambda_l \alpha_{k+1-l} = - \sum_{n=2}^{k+1} \frac{1}{n!} \sum_{\substack{k_1,...,k_n \geq 1 \\ k_1+...+k_n=k+1}} \beta_{k_1} ... \beta_{k_n} . \tag{S94}$$

This yields a function of the α_k that does not depend on the m_{ij}.

Therefore the value of α_k is the same for all circulations, which concludes the proof by induction.

3.4.4 Circulation graphs have the same fixation probability as the well-mixed population

We have shown that for circulation graphs, the extinction probability p_i of one mutant does not depend on the initial deme i. Let us denote it by $\tilde{p}$. Applying Eq. S39 to a circulation graph (which satisfies $\sum_i m_{ij} = \sum_i m_{ji} = 1$) yields

$$\tilde{p} = \exp[\lambda(\tilde{p} - 1)] . \tag{S95}$$

This is the same equation as Eq. S13, which holds for the extinction probability p of one mutant in a well-mixed population. This equation admits a unique solution apart from 1, which entails $p = \tilde{p}$.

Note that rewriting Eq. S76 as

$$\tilde{p} = 1 + \sum_{k=1}^{\infty} \alpha_k x^k \,, \tag{S96}$$

and applying Eq. S78 gives the same result.

Therefore, within the branching process description, all circulation graphs have exactly the same fixation probability as the well-mixed population.

3.5 Circulations have the same average extinction time as a well-mixed population

The average extinction time in a structured population is given by Eq. S31, and that in a well-mixed population is given by a similar formula involving the well-mixed generating function g (see Eq. S13).

Consider a circulation initialized with a single mutant in one deme. In the branching process approximation, the circulation has generating function $\mathbf{h}$, with components $(h_1, ..., h_D)$. Let us show by induction that for all $n \geq 1$, the n-th iterate of $\mathbf{h}$ applied to vector $\mathbf{0} = (0, ..., 0)$ has all its components equal to $g^{(n)}(0)$, which is the n-th iterate of the well-mixed generating function g (see Eq. S13) applied to 0.

- **Order 1:**

For all $i = 1, ..., D$, using Eq. S25 and exploiting the fact that $\sum_j m_{ij} = \sum_j m_{ji} = 1$ for circulations gives:

$$h_i(\mathbf{0}) = \exp\left(-\lambda \sum_j m_{ij}\right) = \exp(-\lambda) = g(0). \tag{S97}$$

- **From order $k-1$ to order k:**

Assume there exists $k > 1$ such that $\mathbf{h}^{(k-1)}(\mathbf{0}) = \left(g^{(k-1)}(0), \ldots, g^{(k-1)}(0)\right)$. Then for all $i = 1, \ldots, D$:

$$(\mathbf{h}^{(k)}(\mathbf{0}))_i = h_i(\mathbf{h}^{(k-1)}(\mathbf{0})) = \exp\left(-\lambda \sum_j m_{ij} g^{(k-1)}(0)\right) = \exp(-\lambda g^{(k-1)}(0)) = g^{(k)}(0). \tag{S98}$$

This concludes the proof by induction. Using this equality in Eq. S31, we find that the average extinction time for a circulation starting from a mutant in any deme i is the same as the average extinction time in the well-mixed population. This holds in the regime of frequent migrations where the sampling is well-described by the Poisson approximation.

3.6 All graphs that are not circulations strictly suppress selection

Consider a mutant introduced uniformly at random in a graph at the bottleneck, i.e. with probability proportional to deme bottleneck sizes. (If all demes start at bottleneck size K on average, as in the main text, this means that a mutant is introduced in an initial deme picked uniformly at random.) Here, we will show that for any connected graph that is not a circulation, the mutant fixation probability is strictly smaller than $2st$, the fixation probability in well-mixed populations and circulations. This means that all graphs that are not circulations strictly suppress selection.

For this, we focus on first order terms of the expansions of the extinction probabilities $p_1, ..., p_D$ in powers of st, namely $a_1, ..., a_D$. For circulation graphs, we have already proven that $a_i = 2\ \forall i$. We show that the average of $a_1, ..., a_D$ weighted by deme bottleneck sizes is always smaller than or equal to 2.

3.6.1 When all demes start at K on average

In this convention, $\sum_{i=1}^{D} m_{ij} = 1$ for all j. Here, a mutant is introduced in an initial deme picked uniformly at random, at the bottleneck. Then, the average extinction probability is obtained to first order in st by computing the arithmetic mean of the a_i.

We start from Eqs. S42 and S43. Eq. S42 shows that $(a_1, ..., a_D)$ is an eigenvector associated to the eigenvalue 1. Such an eigenvector exists, as the migration matrix is a column-stochastic matrix (it has non-negative entries with columns summing to 1), which entails that 1 is its largest eigenvalue. In addition, $a_i \geq 0$ for all i since $p_i < 1$.

We next consider Eq. S43 to specify the values of $a_1, ..., a_D$. Summing this equation over i, we write:

$$\sum_{i=1}^{D} b_i = \sum_{i,j} m_{ij} b_j - 2\sum_{i=1}^{D} a_i + \sum_{i=1}^{D} a_i^2\,,\ \text{i.e.}\ ,$$

$$\sum_{i=1}^{D} b_i = \sum_{j=1}^{D} b_j - 2\sum_{i=1}^{D} a_i + \sum_{i=1}^{D} a_i^2\,,\ \text{i.e.}\ ,$$

$$2\sum_{i=1}^{D} a_i = \sum_{i=1}^{D} a_i^2\,.$$

We now apply Cauchy-Schwarz's inequality to the vectors $A = (a_1, \dots, a_D)$ and $\mathbb{1} = (1, \dots, 1)$, yielding:

$$\left(\sum_{i=1}^{D} a_i\right)^2 \leq D\sum_{i=1}^{D} a_i^2 = 2D\sum_{i=1}^{D} a_i\,, \tag{S99}$$

and therefore

$$\frac{1}{D}\sum_{i=1}^{D} a_i \leq 2\,. \tag{S100}$$

Moreover, Eq. S100 is an equality if and only if vectors A and $\mathbb{1}$ are colinear, in which case all a_is are equal. Eq. S42 then yields $\sum_j m_{ij} = 1$ for all i, hence the graph is a circulation. Thus, all graphs that are not circulations strictly suppress selection.

3.6.2 When all demes contribute by K on average

In this convention, $\sum_{i=1}^{D} m_{ji} = 1$ for all j. The proof in this case is very similar, but we start from Eqs. S49 and S50. In addition, here the bottleneck size of deme i is KC_i, where $C_i = \sum_j m_{ji}$ is the sum of incoming migrations to deme i (none of them are equal to 0, since no deme is disconnected). Thus, the average extinction probability is obtained to first order in st by computing the average of the values of a_i weighted by C_i/D.

First, let us make sure that matrix μ, with elements $\mu_{ij} = m_{ij}/C_i$, has 1 as eigenvalue, thereby ensuring that Eq. S49 has a non-trivial solution. For this, we show that its spectrum is the same as that of another matrix whose columns sum to 1. Indeed, let η be an eigenvalue of μ, and y an associated eigenvector. We then have $\forall i \in \{1, ..., D\}$:

$$\eta\, y_i = \sum_{j=1}^{D} \mu_{ij} y_j\,,\ \text{i.e.}\ , \tag{S101}$$

$$\eta\, y_i = \sum_{j=1}^{D} \frac{m_{ij}}{C_i} y_j\,,\ \text{i.e.}\ , \tag{S102}$$

$$\eta\, y_i C_i = \sum_{j=1}^{D} \frac{m_{ij}}{C_j} y_j C_j. \tag{S103}$$

Thus, η is also an eigenvalue of matrix $\tilde{\mu}$ defined by $\tilde{\mu}_{ij} = m_{ij}/C_j$. This entails that μ and $\tilde{\mu}$ share the same spectrum. But $\tilde{\mu}$ is non-negative with columns summing to 1, and therefore 1 is indeed an eigenvalue (the largest).

We now want to characterize eigenvector $(a_1, ..., a_D)$ starting from Eq. S50:

$$b_i = \sum_{j=1}^{D} \frac{m_{ij}}{C_i} b_j - 2a_i + a_i^2 \text{ , i.e. ,} \tag{S104}$$

$$C_i b_i = \sum_{j=1}^{D} \frac{m_{ij}}{C_j} C_j b_j - 2C_i a_i + C_i a_i^2 \,. \tag{S105}$$

Summing over i on both sides, and recalling that $\sum_i m_{ij} = C_j$, we obtain

$$\sum_{i=1}^{D} C_i b_i = \sum_{j=1}^{D} C_j b_j - 2\sum_{i=1}^{D} C_i a_i + \sum_{i=1}^{D} C_i a_i^2 \text{ , i.e. ,} \tag{S106}$$

$$2\sum_{i=1}^{D} C_i a_i = \sum_{i=1}^{D} C_i a_i^2 \,. \tag{S107}$$

Applying Cauchy-Schwarz's inequality to the vectors $(\sqrt{C_1}, \dots, \sqrt{C_D})$ and $(\sqrt{C_1}a_1, \dots, \sqrt{C_D}a_D)$ yields

$$\left(\sum_{i=1}^{D} C_i a_i\right)^2 \leq \sum_{i=1}^{D} C_i \sum_{j=1}^{D} C_j a_j^2 = 2\sum_{i=1}^{D} C_i \sum_{j=1}^{D} C_j a_j \,. \tag{S108}$$

Since $\sum_{i=1}^{D} C_i = D$, we obtain

$$\left(\sum_{i=1}^{D} C_i a_i\right)^2 \leq 2D\sum_{i=1}^{D} C_i a_i \,, \tag{S109}$$

which yields the desired result:

$$\sum_{i=1}^{D} \frac{C_i}{D} a_i \leq 2 \,. \tag{S110}$$

Eq. S110 is an equality if and only if $(\sqrt{C_1}, \dots, \sqrt{C_D})$ and $(\sqrt{C_1}a_1, \dots, \sqrt{C_D}a_D)$ are colinear. In that case, all a_is are equal to 2, and Eq. S49 yields $\sum_j \mu_{ij} = 1$ for all i, hence the graph is a circulation.

To summarize, we have shown that in the branching process approach, and if migration probabilities are all of order 1 (which allows to use the Poisson distribution), any graph that is not a circulation strictly suppresses natural selection to first order in st. In contrast, circulation graphs have the same fixation probability as a well-mixed population.

4 Structured populations on graphs with serial dilutions: branching process with binomial sampling

4.1 Motivation

Recall that upon the first dilution-migration step, we sample on average $Km_{ij}x_0'^{(i)}$ mutants that go to deme j using binomial sampling from the grown deme i. The Poisson approximation made above requires $\lambda_{ij} = Km_{ij}x_0'^{(i)}$ to be of order 1. However, this does not hold if m_{ij} is of order st or smaller. Moreover, the identification of terms in expansions in powers of st that we performed above to obtain extinction probabilities has to be handled differently for these small m_{ij}. To treat the case of rarer migrations, of order st or $(st)^2$, we thus need to generalize our treatment and to go back to the complete binomial law. However, we will remain within the multi-type branching process approximation, assuming $s > 0$, $st \ll 1$, $K \gg 1$ and $Kst \gg 1$.

First, we will treat the convention $\sum_i m_{ij} = 1$, i.e. all demes start at K on average. The corresponding results are shown in the main text. Then, we will discuss how these calculations can be extended to the other convention $\sum_j m_{ij} = 1$, where all demes contribute by K on average, and show the corresponding results.

4.2 When all demes start at K on average

4.2.1 Generating function

The generating function $\mathbf{f}(\mathbf{x})$ is given by Eq. S23. Starting from one mutant in deme i, with deme size equal to N_i' and mutant ratio $x_0'^{(i)}$ after growth, the number of mutants sent from i to j is sampled from the binomial law $\mathcal{B}\left(N_i', Kx_0'^{(i)} m_{ij}/N_i'\right)$. Therefore

$$\phi_i(r_1, ..., r_D) = \prod_{j=1}^{D} \binom{N_i'}{r_j} \left(\frac{K}{N_i'} x_0'^{(i)} m_{ij}\right)^{r_j} \left(1 - \frac{K}{N_i'} x_0'^{(i)} m_{ij}\right)^{N_i' - r_j} . \tag{S111}$$

The i-th component of the generating function now reads

$$f_i(\mathbf{x}) = \prod_{j=1}^{D} \left(1 - (1 - x_j)\frac{\lambda}{N_i'} m_{ij}\right)^{N_i'} , \tag{S112}$$

where λ is given by Eq. S12.

One can consider a well-mixed population with sampling performed exactly as in our structured populations on graphs (note that this is slightly different from the case considered in Section 1 where bottleneck size was exactly K). This is useful as reference. Then, the (single-type) generating function reads

$$f(x) = \left(1 - (1 - x)\frac{\lambda}{N'}\right)^{N'} , \tag{S113}$$

where N' is the size of the population after growth.

4.2.2 Extinction probabilities: general expansion

Let p_i be the extinction probability starting from one mutant in deme i. Under the assumptions $s > 0$, $st \ll 1$, $K \gg 1$ and $Kst \gg 1$, let us expand p_i perturbatively in powers of st:

$$p_i = 1 - a_i st + \frac{b_i}{2}(st)^2 - \frac{c_i}{6}(st)^3 + o((st)^3) . \tag{S114}$$

The vector of extinction probabilities $\mathbf{p} = (p_i, ..., p_D)$ is a fixed point of the generating function. For each component i, we expand pertubatively in powers of st the equality

$$p_i = f_i(\mathbf{p}) . \tag{S115}$$

Note that $(1 - p_j)\lambda m_{ij}$ is at most of order st (if m_{ij} is of order one). Furthermore, since $N_i' > K$ for all i, and $st \gg 1/K$, we have $st \gg 1/N_i'$. We can then expand the generating function components as

$$\begin{aligned} f_i(\mathbf{p}) = \prod_{j=1}^{D} \Bigg[& 1 - (1 - p_j)\lambda m_{ij} + \frac{N_i'(N_i' - 1)}{2} \left((1 - p_j)\frac{\lambda}{N_i'} m_{ij}\right)^2 \\ & - \frac{N_i'(N_i' - 1)(N_i' - 2)}{6} \left((1 - p_j)\frac{\lambda}{N_i'} m_{ij}\right)^3 + o((st)^3) \Bigg] \\ = \prod_{j=1}^{D} \Bigg[& 1 - (1 - p_j)\lambda m_{ij} + \frac{1}{2}\left((1 - p_j)\lambda m_{ij}\right)^2 - \frac{1}{6}\left((1 - p_j)\lambda m_{ij}\right)^3 + o((st)^3) \Bigg] . \end{aligned} \tag{S116}$$

To further expand Eq. S116, we need to specify how migration probabilities scale with st. Below, we consider different regimes.

Note that the i-th component of the generating function in Eq. S112 can be written as

$$f_i(\mathbf{x}) = \prod_{j=1}^{D} \exp\left[N_i' \log\left(1 - (1 - x_j)\frac{\lambda}{N_i'}\right)\right] . \tag{S117}$$

If we take the limit $N'_i \to \infty$, then for any k, $(st)^k \ll 1/N'_i$. Applying f_i to $\mathbf{p}$, expanding the logarithm in the powers of st and discarding terms of order $1/N'_i$ then yields

$$f_i(\mathbf{p}) = \exp\left[\lambda \sum_{j=1}^{D} m_{ij}(p_j - 1)\right] , \tag{S118}$$

which is the same generating function as the one we obtained with the Poisson approximation in Eq. S25. This is consistent, since the Poisson approximation requires $N'_i \to \infty$. However, for large but finite populations, one should assume that N'_i is finite, meaning that $(st)^k \ll 1/N'_i$ will break down beyond some k. For this reason, we present a detailed analysis of the binomial case, restricting to the first terms of the expansion in st, detailed in Eq. S116.

4.2.3 Well-mixed population

For a well-mixed population, the probability of extinction $p = 1 - ast + b(st)^2/2 + o((st)^2)$ of the mutant satisfies the equation $p = f(p)$ with f defined in Eq. S113. Expanding this equation in powers of st and identifying terms yields $a = 2$ and $b = 10/3$. This result coincides with the one obtained in Section 1, despite the minor difference in sampling. Note that the Poisson approximation holds in the well-mixed case.

4.2.4 When all migration probabilities are of order 1

Assuming that all migration probabilities are of order 1, for all i, $f_i(\mathbf{p})$ in Eq. S116 can be expanded up to third order in st as:

$$\begin{aligned} f_i(\mathbf{p}) = \prod_{j=1}^{D} \Big[& 1 - m_{ij}a_j st + \frac{m_{ij}}{2}\left(b_j - 2a_j + m_{ij}a_j^2\right)(st)^2 \\ & - \frac{m_{ij}}{6}\left(c_j - 3b_j + 3a_j + 3m_{ij}a_jb_j - 6m_{ij}a_j^2 + m_{ij}^2a_j^3\right)(st)^3 \Big] . \end{aligned} \tag{S119}$$

Using the expansion of p_i in Eq. S114, identifying the first and second order terms in st in the equality $p_i = f_i(\mathbf{p})$ yields:

$$a_i = \sum_j m_{ij}a_j , \tag{S120}$$

$$b_i = \sum_j m_{ij}b_j - 2a_i + a_i^2 . \tag{S121}$$

These equations match those we obtained with the Poisson law. Again, thanks to the Cauchy-Schwartz inequality, we show that $\sum_i a_i/D \leq 2$, and the case of equality corresponds to $a_i = 2$ for all i. Then, $\sum_j m_{ij} = 1$ for all j, therefore the graph is a circulation. Hence, suppression is strict to first order in st for all graphs that are not circulations and where migration probabilities are all of order 1.

Identifying the third order terms in st in the equality $p_i = f_i(\mathbf{p})$ further yields

$$c_i = \sum_j m_{ij}c_j - 3b_i + 3a_ib_i - 3a_i + 3a_i^2 - 2a_i^3 . \tag{S122}$$

If the graph is a circulation, then by the same argument detailed in 3.4.3, using the Jordan normal form of the migration matrix, we obtain $b_i = 10/3$ for all i. This is consistent with the second order coefficient obtained using the Poisson approximation for circulation graphs in 3.3.1, and coincides with the well-mixed population result.

4.2.5 When exchanges between different demes are of order st

This time, assume that migration probabilities between different demes are of order st, while self-loops can have a dominant term of order 1. We write

$$m_{ij} = m_{ij}^{(1)} st \;\text{ if } i \neq j , \tag{S123}$$

$$m_{ii} = 1 + m_{ii}^{(1)} st . \tag{S124}$$

Expanding Eq. S116 yields

$$a_i = -\sum_j a_j m_{ij}^{(1)} + \frac{a_i^2}{2}\,, \tag{S125}$$

$$0 = -3a_i + 3a_i^2 - 2a_i^3 - 3b_i + 3a_i b_i - 3\sum_j b_j m_{ij}^{(1)}\,. \tag{S126}$$

From $\sum_i m_{ij} = 1$, we deduce $\sum_i m_{ij}^{(1)} = 0$. Summing the two equations above on i, we can again apply the Cauchy-Schwartz inequality to get $\sum_i a_i/D \leq 2$. Note that the case of equality gives $a_i = 2$ for all i, and then the second equation simplifies to $\sum_i b_i/D = 10/3$. In that case, the second order term is the same starting from any deme ($b_i = 10/3$ for all i) if the graph is a circulation.

4.2.6 When exchanges between different demes are of order $(st)^2$

This time, we write

$$m_{ij} = m_{ij}^{(2)}(st)^2 \quad \text{if } i \neq j\,, \tag{S127}$$

$$m_{ii} = 1 + m_{ii}^{(2)}(st)^2\,. \tag{S128}$$

Expanding Eq. S116 yields

$$a_i = 2\,, \tag{S129}$$

$$0 = 3a_i - 6a_i^2 + a_i^3 - 3b_i + 3a_i b_i + 6\sum_j a_j m_{ij}^{(2)}\,, \tag{S130}$$

and the second term becomes

$$b_i = \frac{10}{3} - 4\sum_j m_{ij}^{(2)}. \tag{S131}$$

Summing on i and using $\sum_i m_{ij}^{(2)} = 0$ yields

$$\frac{1}{D}\sum_i b_i = \frac{10}{3}\,. \tag{S132}$$

Thus, in this case, the fixation probability is the same as in the well-mixed case up to first order. Furthermore, the fixation probability averaged over all starting demes is the same as in the well-mixed case up to second order. Note that Eq. S131 entails that the second order term is the same starting from any deme ($b_i = 10/3$ for all i) if and only if the graph is a circulation.

4.2.7 Application to the star graph

We apply the results from the three previous subsections to the star graph (see Fig. 1), with $D = 5$ demes, $K = 1000$ individuals per deme on average at the bottlenecks, and a migration asymmetry $\alpha = m_I/m_O$. We compute the first order term a_C (resp. a_L, see Section 3.3.2) of the fixation probability of one mutant starting in the center (resp. starting in a leaf), in different regimes of migration probabilities. Fig. S1 shows that the branching process predictions agree very well our stochastic simulations. In particular, we see that starting from the center for $\alpha > 1$ amplifies natural selection for frequent migrations, while starting from the leaf suppresses selection. The opposite holds for $\alpha < 1$: starting from a leaf amplifies selection while starting from the center suppresses it. Averaging over the initial position of the mutant always results in suppression, as shown in Fig. 2.

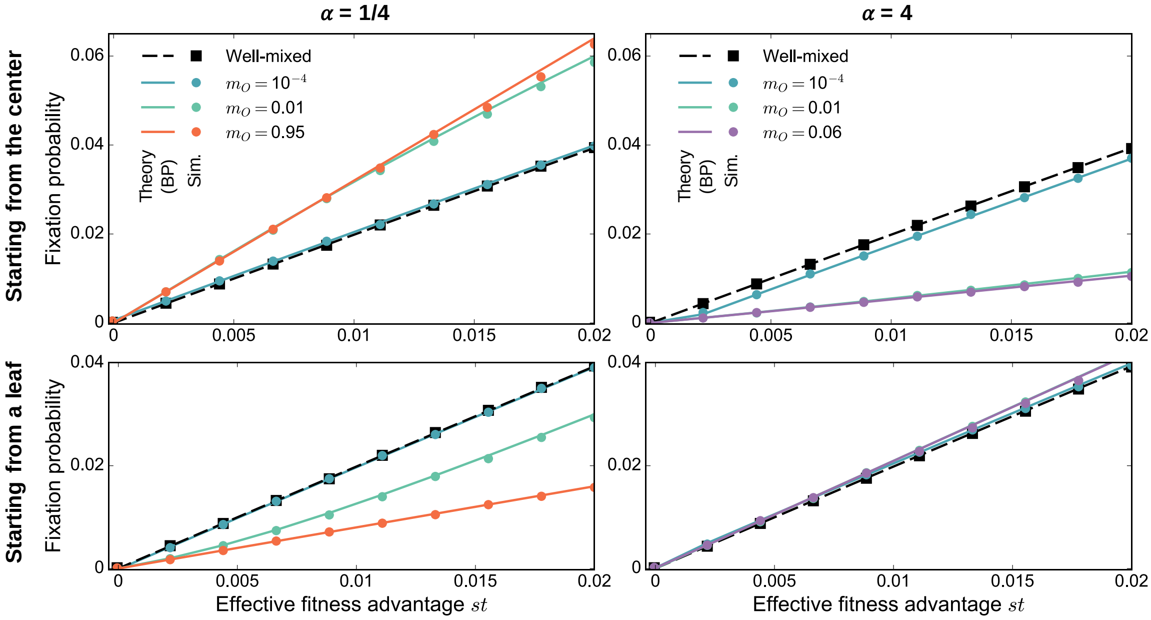

Figure S1: **Mutant fixation in the star starting from the center or from a leaf.** Mutant fixation probability is plotted versus the effective fitness advantage st of the mutant. We consider a star with $D = 5$ demes, and $K = 1000$ individuals per deme on average at the bottleneck, for migration asymmetries $\alpha = 1/4$ (left) and $\alpha = 4$ (right). The growth phase duration is $t = 5$. We start with one mutant of fitness $f_M = 1 + s$ placed in the center (top) or in one of the leaves (bottom), all other individuals being wild-types with fitness $f_W = 1$. Markers are simulation results ("Sim.") averaged on 1 million realizations. Lines are theoretical predictions obtained through a branching process ("BP") approach. The well-mixed case is shown for comparison, with simulations performed for a well-mixed population with $KD = 5000$ individuals at the bottleneck, initialized with one mutant.

4.2.8 Numerical results on fixation dynamics

While we obtained results on fixation probabilities and extinction times, another important question regards the dynamics of fixation. Does spatial structure impact how fast fixation occurs? We addressed this question in numerical simulations.

Fig. S2 shows the growth of mutant fraction in trajectories that end in mutant fixation, starting from one mutant in different spatial structures. For the migration asymmetry chosen, which leads to strong suppression of selection (see Fig. 2), we observe that mutant fraction grows faster in the star with frequent migrations than in the well-mixed population. This is associated to the suppression of selection and to the acceleration of extinction obtained in this case (see Fig. 2). Meanwhile, the clique with frequent migrations behaves like the well-mixed population, corroborating their similarities shown for fixation probabilities and for extinction times (see Section 3.5). For less frequent migrations, the growth of mutant fraction is slower in structured populations (clique and star) than in the well-mixed population: in this regime, spatial structure slows down the dynamics.

Fig. S3 shows that, for frequent migrations, the average mutant fixation time is smaller in the star than in the well-mixed population. This further confirms the acceleration of the dynamics observed in this regime, in Fig. 2 for extinction times, and in Fig. S2 for the growth of mutant fraction in trajectories yielding fixation.

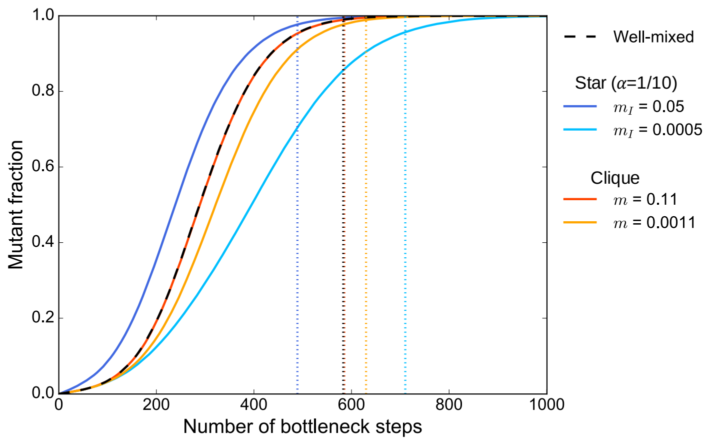

Figure S2: **Mutant fraction growth leading to fixation**. The mutant fraction in the total population is shown versus the number of bottleneck steps for the well-mixed population, the star and the clique in different migration regimes. We consider a star with $D = 5$ demes, and $K = 1000$ individuals per deme on average at the bottleneck, for migration asymmetry $\alpha = 1/10$, leading to strong suppression of selection (see Fig. 2). We also consider a clique with the same number of demes of the same size. The well-mixed case is shown for comparison, with $KD = 5000$ individuals at the bottleneck. In all cases, we start with one mutant of fitness $f_M = 1 + s$ placed uniformly at random at a bottleneck, all other individuals being wild-types with fitness $f_W = 1$. The growth phase duration is $t = 5$, and the effective fitness advantage is set to $st = 0.2$. Curves represent simulation results, averaged over 2000 to 5000 fixation trajectories. Vertical dotted lines show the average fixation times for these structures. Note that those corresponding to the clique with $m = 0.11$ and to the star are superposed.

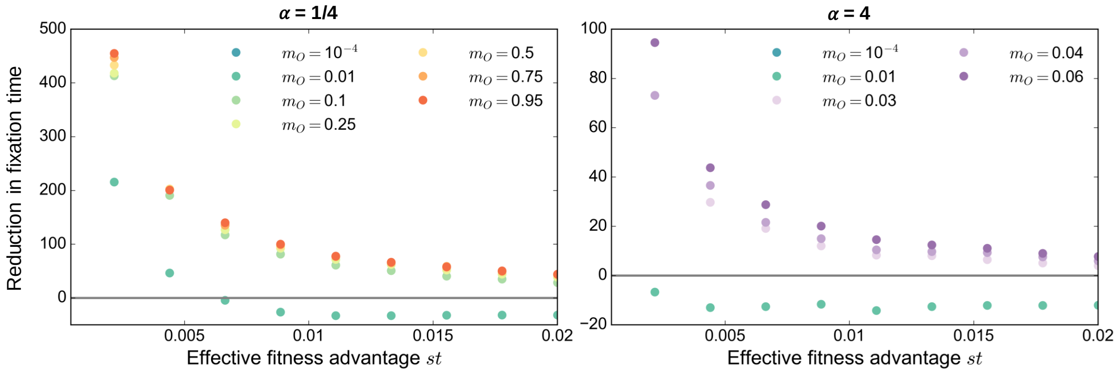

Figure S3: **Reduction of the average mutant fixation time in the star.** The difference between the average fixation time (in numbers of dilution steps) in the well mixed population and that in the star is plotted versus the effective fitness advantage st of the mutant. As in Fig. 2, we consider a star with $D = 5$ demes, and $K = 1000$ individuals per deme on average at the bottleneck, for migration asymmetries $\alpha = 1/4$ (left) and $\alpha = 4$ (right). We start with one mutant of fitness $f_M = 1 + s$ placed uniformly at random at a bottleneck, all other individuals being wild-types with fitness $f_W = 1$. The growth phase duration is $t = 5$. Markers represent simulation results averaged over 100 000 realizations. The well-mixed case, taken as reference, corresponds to a population with $KD = 5000$ individuals at the bottleneck, initialized with one mutant.

4.3 When all demes contribute by K on average

The previous subsections focused on the case where all demes have the same average bottleneck size K, i.e. $\sum_j m_{ji} = 1$ for all i. The branching process approximation can also be used in the case where all demes contribute by K individuals on average to each bottleneck, but can have different average

bottleneck sizes. In that case, $\sum_j m_{ij} = 1$ for all i. The generating function's i-th component keeps the form of Eq. S112, but migrating probabilities m_{ij} are replaced by $\mu_{ij} = m_{ij}/\sum_k m_{ki}$, as in the previous section:

$$f_i(\mathbf{x}) = \prod_{j=1}^{D} \left(1 - (1 - x_j)\frac{\lambda}{N_i'}\mu_{ij}\right)^{N_i'} . \tag{S133}$$

All the calculations performed assuming $\sum_j m_{ji} = 1$ for all i can be adapted simply replacing m_{ij} by μ_{ij}, yielding predictions for fixation probabilities and average fixation time for different graph structures.

In Fig. S4, we show both analytical predictions and simulation results for the star graph with $D = 5$ and equally contributing demes. Note that corresponding results in the case where all demes start at the same bottleneck size on average are shown in Fig. 2.

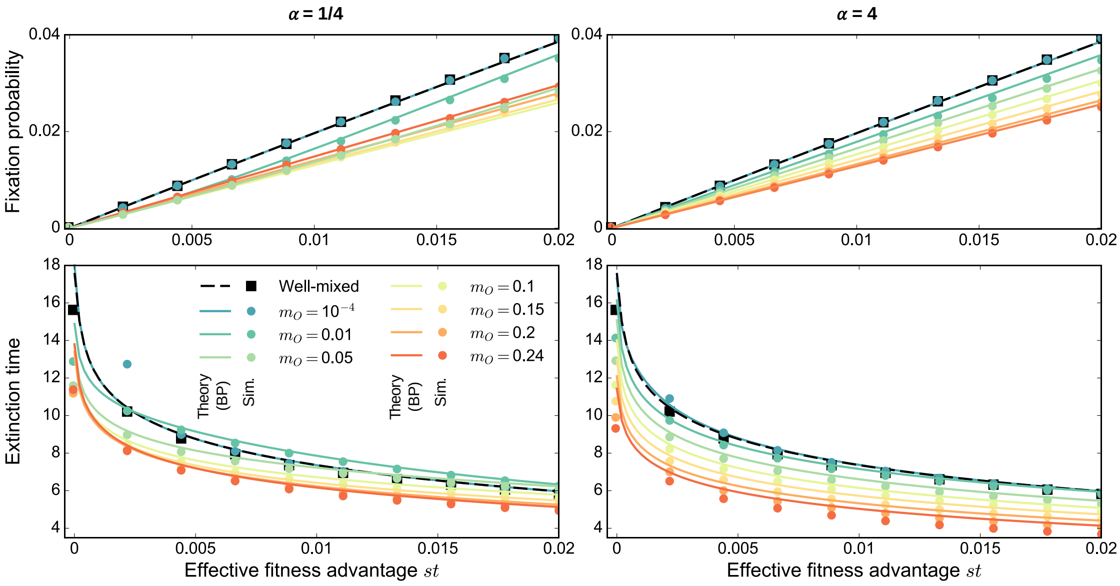

Figure S4: **Mutant fixation in the star when all demes contribute by K on average.** Mutant fixation probability (top) and average extinction time (bottom, in numbers of dilution steps) are plotted versus the effective fitness advantage st of the mutant. We consider a star with $D = 5$ demes, and all demes contribute on average by $K = 1000$ individuals to each bottleneck. Migration asymmetries are $\alpha = 1/4$ (left) and $\alpha = 4$ (right). We start with one mutant of fitness $f_M = 1 + s$ placed uniformly at random at a bottleneck, all other individuals being wild-types with fitness $f_W = 1$. The growth phase duration is $t = 5$. Markers represent simulation results ("Sim."), averaged on 1 million realizations. Lines are theoretical predictions from our branching process ("BP") approach. The well-mixed case is shown for comparison, with simulations performed for a well-mixed population with $KD = 5000$ individuals at the bottleneck, initialized with one mutant. The convention $\sum_j m_{ij} = 1$ constrains the value of m_O to be between 0 and 1/4.

5 Different models for serial dilution in structured populations

Several models can be considered to describe migration and dilution in a structured population. Here, we define three of them, which rely on distinct sampling schemes which involve different fluctuations in the number of individuals that compose each new bottleneck state. This could affect the evolutionary dynamics. Let us compare these models, in the convention used in the main text where demes have the same average size K at the bottleneck, i.e. $\sum_j m_{ji} = 1$ for all i.

Binomial samplings for migration and dilution. This model is the main one studied in this paper, and is detailed in the Methods in the main text. Starting from D demes with mutant fractions $x_1, \dots, x_D$, we begin with a growth phase at the end of which the mutant ratios become $x'_1, \dots, x'_D$ in demes of large sizes $N'_1, \dots, N'_D$. Then, we proceed with the dilution and migration step by drawing the number of migrating mutants (resp. wild-types) from deme i to j in a binomial distribution with N'_i trials and probability of success $K/N'_i x'_i m_{ij}$ (resp. $K/N'_i(1 - x'_i)m_{ij}$). This model is based on independent binomial samplings for each type, for any migration itinerary. The bottleneck size of each deme fluctuates around the average value K.

Multinomial samplings for migration and dilution. A variant uses multinomial samplings at the migration and dilution step, after the growth phase. For any deme i, the numbers of incoming individuals from all demes (including mutants and wild-types) are sampled from a multinomial distribution with K trials and probabilities $(x'_1 m_{1i}, (1 - x'_1)m_{1i}, \dots, x'_D m_{Di}, (1 - x'_D)m_{Di})$. Note that these probabilities sum to 1 since $\sum_j m_{ji} = 1$. In this model, the deme sizes are exactly equal to K at bottleneck. Multinomial samplings are performed independently for each deme. Applied to a well-mixed population, this model reduces to the Wright-Fisher model.

Local binomial samplings for dilution, after migration. In this model, migrations are performed as a deterministic step, before sampling, exactly as in a structured Wright-Fisher model [9]. After the growth phase, the mutant ratios are $x'_1, \dots, x'_D$. After migration (and before dilution), the local mutant ratios become $\tilde{x}_1, \dots, \tilde{x}_D$ where $\tilde{x}_i = \sum_j m_{ji} x'_j$ for all i. Note that this migration step does not take into account the actual sizes of the demes, essentially assuming that they are all infinite. Then, dilution is performed locally in each deme by sampling the number of mutant individuals present in deme i at the next bottleneck from a binomial distribution with K trials and probability of success $\tilde{x}_i$. The number of wild-types in deme i at the next bottleneck is then taken so that deme i has exactly size K. Applied to a well-mixed population, this model also reduces to the Wright-Fisher model.

Comparison. The branching process approach can be applied to these three models. Under the assumption $st \gg 1/K$, we find that the first three order terms in the perturbative expansion in st of the generating function, detailed for the first model in Eq. S112, are the same for all models. Therefore, all those models should yield the same results in the domain of validity of the branching process analysis. This is indeed confirmed by simulations, as shown in Fig. S5.

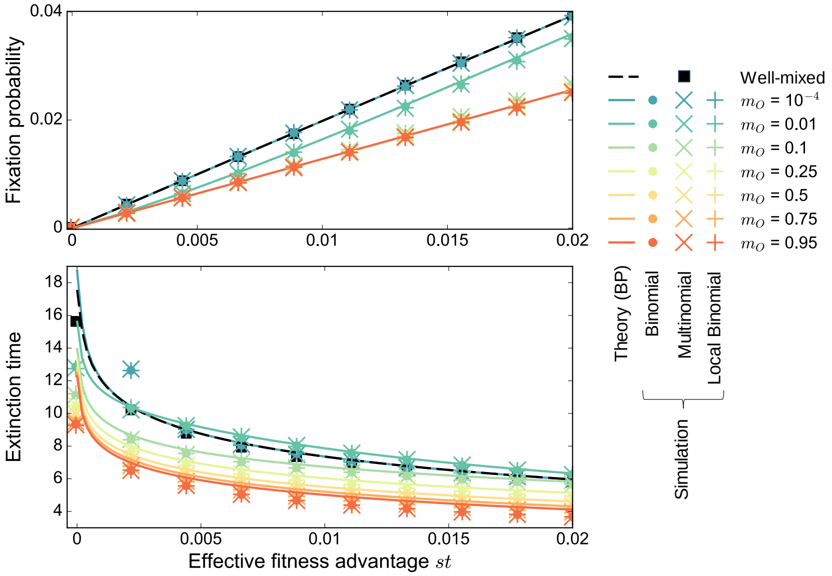

Figure S5: **Mutant fixation in the star for different models of serial dilution.** Mutant fixation probability (up) and average extinction time (bottom, in numbers of dilution steps) are plotted versus the effective fitness advantage st of the mutant. We consider a star with $D = 5$ demes, and $K = 1000$ individuals per deme at the bottleneck (either exactly or on average depending on the model), for migration asymmetry $\alpha = 1/4$. The growth phase duration is $t = 5$. We start with one mutant of fitness $f_M = 1 + s$ placed uniformly at random at a bottleneck, all other individuals being wild-types with fitness $f_W = 1$. Markers are simulation results ("Sim.") obtained from 1 million realizations. The three different markers correspond to three different models defined in Section 5, that use binomial samplings for each migration itinerary and each type ("Binomial") , multinomial samplings for each deme ("Multinomial"), or binomial samplings for each deme after a deterministic migration step ("Local Binomial") . Lines are theoretical predictions obtained through a branching process ("BP") approach. The well-mixed case is shown for comparison, with simulations performed for a well-mixed population with $KD = 5000$ individuals at the bottleneck, initialized with one mutant.

6 Dirichlet cliques

To go beyond graphs with high symmetry like the clique and the star, we consider graphs where migration probabilities are drawn in Dirichlet distributions. Indeed, using these probability distributions ensure that our migration probabilities satisfy $\sum_i m_{ij} = 1$ for all i, and allow us to tune migration asymmetry. Here, we give the definition of the Dirichlet distribution, as well as some examples, and we explain how we use it to generate migration probabilities.

6.1 Definition of the Dirichlet distribution

The Dirichlet distribution of order $D \geq 2$ with parameters $\eta_1, ..., \eta_D$ has the following probability density function for $\mathbf{x} = (x_1, ..., x_D) \in [0, 1]^D$ in the $D - 1$ simplex (such that $\sum_i x_i = 1$):

$$\Phi_{(\eta_1,...,\eta_D)}(\mathbf{x}) = \frac{1}{B(\eta_1, ..., \eta_D)} \prod_{i=1}^{D} x_i^{\eta_i - 1} , \tag{S134}$$

where B is the Euler beta function, which can be expressed in terms of the gamma function Γ as

$$B(\eta_1, ..., \eta_D) = \frac{\prod_{i=1}^{D} \Gamma(\eta_i)}{\Gamma\left(\sum_{i=1}^{D} \eta_i\right)} . \tag{S135}$$

The parameters $\eta_1, ..., \eta_D$ impact both the mean values of the sampled variables and their variances and covariances. Indeed, random variables $X_1, ..., X_D$ sampled from the Dirichlet distribution have

the following means and covariances:

$$\mathbb{E}[X_i] = \tilde{\eta}_i \tag{S136}$$

$$\mathrm{Var}[X_i] = \frac{\tilde{\eta}_i(1-\tilde{\eta}_i)}{\sum_j \eta_j + 1} \tag{S137}$$

$$\mathrm{Cov}[X_i, X_j] = \frac{-\tilde{\eta}_i\tilde{\eta}_j}{\sum_j \eta_j + 1} \quad \text{for } i \neq j\,, \tag{S138}$$

where $\tilde{\eta}_i = \eta_i / \sum_j \eta_j$. Thus, on average, the higher η_i is with respect to the other coefficients η_j with $j \neq i$, the closer x_i is to one. Moreover, as the sum $\sum_j \eta_j$ of the coefficients η_j grows, the variances and covariances of the variables $X_1, ..., X_D$ decrease.

6.2 Examples of Dirichlet distributions

When all parameters are equal. If all parameters $\eta_1, ..., \eta_D$ are equal to η, then $X_1, ..., X_D$ all have the same mean $1/D$. When $\eta = 1$, the distribution is uniform on the simplex. If η grows above 1, the variances and covariances of $X_1, ..., X_D$ decrease, and their probability density concentrates around the mean. Conversely, as η becomes smaller, variances grow and the probability density concentrates around small and large values of $x_1, ..., x_D$, near the simplex boundary. Fig. S6 illustrates the impact of increasing η for $D = 3$, where the random variables X_1, X_2, X_3 have average value $1/3$.

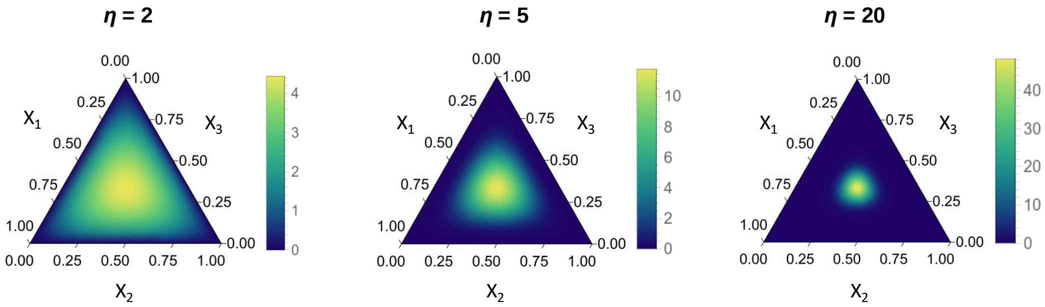

Figure S6: Probability density function of random variables (X_1, X_2, X_3) sampled from a Dirichlet distribution with parameters all equal to η. The probability density function is represented on a ternary plot, where point coordinates are obtained by projecting along lines parallel to the triangle edges. The mean values of X_1, X_2, X_3 are always equal to $1/3$, and the density concentrates around the central point as η grows.

When one parameter is larger than others. Consider a Dirichlet distribution of parameters $\eta_1 \equiv \eta_0$, $\eta_2 = 1$, ..., $\eta_D = 1$ with $\eta_0 > 1$. The variables $X_1, ..., X_D$ have mean values

$$\mathbb{E}[X_1] = \frac{\eta_0}{\eta_0 + D - 1}\,, \tag{S139}$$

$$\mathbb{E}[X_j] = \frac{1}{\eta_0 + D - 1} \quad \text{for } j \geq 2\,. \tag{S140}$$

As η_0 becomes larger, the mean of X_1 grows towards 1, while the mean of X_j for $j > 1$ decreases towards 0. If $\eta_0 > D - 1$, when η_0 grows, the variances of $X_1, ..., X_D$ decrease towards 0, while their covariances increase towards 0. Therefore, the probability density concentrates around the mean values. An example is shown for $D = 3$ in Fig. S7.

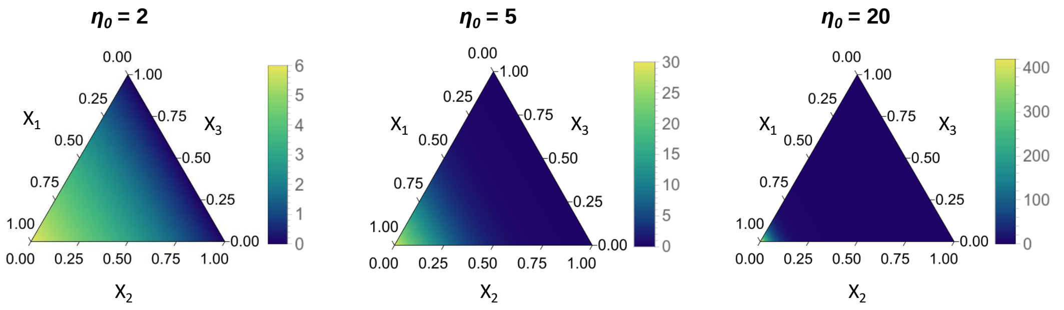

Figure S7: Ternary plot of the probability density function of random variables (X_1, X_2, X_3) sampled from a Dirichlet distribution with parameters $(\eta_0, 1, 1)$. As η_0 grows, the probability density concentrates around the left corner, which corresponds to $(x_1, x_2, x_3) = (1, 0, 0)$.

6.3 Application: Dirichlet cliques

In Fig. 3, for $D = 5$, we generated graphs with migration probabilities drawn in Dirichlet distributions, which we call Dirichlet cliques. For any deme j, we sampled the incoming migration probabilities $(m_{1j}, m_{2j}, m_{3j}, m_{4j}, m_{5j})$ from a Dirichlet distribution.

If all demes are equivalent (top panel of Fig. 3), we take all Dirichlet parameters equal to η. All migration probabilites then have mean $1/5$. Sampling a Dirichlet clique with η very large yields a Dirichlet clique very close to the standard clique, with $m = 1/5$.

If deme 1 is advantaged (bottom panel of Fig. 3), then for any deme j, the incoming migration probabilities $(m_{1j}, m_{2j}, m_{3j}, m_{4j}, m_{5j})$ are sampled from a Dirichlet distribution with parameters $(\eta_0, 1, 1, 1, 1)$. This means that all demes are more likely to receive migrants from deme 1 than from the others.

References

[1] M. Manhart, B. V. Adkar, and E. I. Shakhnovich. Trade-offs between microbial growth phases lead to frequency-dependent and non-transitive selection. *Proceedings of the Royal Society B: Biological Sciences*, 285(1872):20172459, 2018.

[2] J. F. Crow and M. Kimura. *An Introduction to Population Genetics Theory*. Blackburn, 2009.

[3] M. Kimura and T. Ohta. The average number of generations until fixation of a mutant gene in a finite population. *Genetics*, 61(3):763–771, 03 1969.

[4] T. E. Harris. *The Theory of Branching Processes*. Springer, 1963.

[5] J. B. Haldane. A mathematical theory of natural and artificial selection. V. Selection and mutation. *Camb. Philos. Soc.*, 23:838–844, 1927.

[6] L. Marrec, I. Lamberti, and A.-F. Bitbol. Toward a universal model for spatially structured populations. *Physical Review Letters*, 127(21):218102, 2021.

[7] H. K. Alexander. Conditional distributions and waiting times in multitype branching processes. *Advances in Applied Probability*, 45(3):692–718, 2013.

[8] E. Lieberman, C. Hauert, and M. A. Nowak. Evolutionary dynamics on graphs. *Nature*, 433(7023):312–316, 2005.

[9] C. Burden and R. Griffiths. Stationary distribution of a 2-island 2-allele Wright-Fisher diffusion model with slow mutation and migration rates. *Theoretical Population Biology*, 124, 02 2018.
